# Chloroplastic Sec14-like proteins modulate growth and phosphate deficiency responses in *Arabidopsis* and rice

**DOI:** 10.1093/plphys/kiad212

**Published:** 2023-04-06

**Authors:** Mailun Yang, Yasuhito Sakruaba, Toshiki Ishikawa, Namie Ohtsuki, Maki Kawai-Yamada, Shuichi Yanagisawa

**Affiliations:** Agro-Biotechnology Research Center, Graduate School of Agricultural and Life Sciences, The University of Tokyo, Tokyo 113-8657, Japan; Agro-Biotechnology Research Center, Graduate School of Agricultural and Life Sciences, The University of Tokyo, Tokyo 113-8657, Japan; Graduate School of Science and Engineering, Saitama University, Saitama 338-8570, Japan; Agro-Biotechnology Research Center, Graduate School of Agricultural and Life Sciences, The University of Tokyo, Tokyo 113-8657, Japan; Graduate School of Science and Engineering, Saitama University, Saitama 338-8570, Japan; Agro-Biotechnology Research Center, Graduate School of Agricultural and Life Sciences, The University of Tokyo, Tokyo 113-8657, Japan

## Abstract

Phosphorus is an essential nutrient acquired from soil as phosphate (Pi), and its deficiency severely reduces plant growth and crop yield. Here, we show that single nucleotide polymorphisms (SNPs) at the *PHOSPHATIDYLINOSITOL TRANSFER PROTEIN7* (*AtPITP7*) locus, which encodes a chloroplastic Sec14-like protein, are associated with genetic diversity regarding Pi uptake activity in *Arabidopsis* (*Arabidopsis thaliana*). Inactivation of *AtPITP7* and its rice (*Oryza sativa*) homolog (*OsPITP6*) through T-DNA insertion and CRISPR/Cas9-mediated gene editing, respectively, decreased Pi uptake and plant growth, regardless of Pi availability. By contrast, overexpression of *AtPITP7* and *OsPITP6* enhanced Pi uptake and plant growth, especially under limited Pi supply. Importantly, overexpression of *OsPITP6* increased the tiller number and grain yield in rice. Targeted metabolome analysis of glycerolipids in leaves and chloroplasts revealed that inactivation of *OsPITP6* alters phospholipid contents, independent of Pi availability, diminishing the reduction in phospholipid content and increase in glycolipid content induced by Pi deficiency; meanwhile, overexpression of *OsPITP6* enhanced Pi deficiency-induced metabolic alterations. Together with transcriptome analysis of *ospitp6* rice plants and phenotypic analysis of grafted *Arabidopsis* chimeras, these results suggest that chloroplastic Sec14-like proteins play an essential role in growth modulations in response to changes in Pi availability, although their function is critical for plant growth under any Pi condition. The superior traits of *OsPITP6*-overexpressing rice plants also highlight the potential of OsPITP6 and its homologs in other crops as additional tools for improving Pi uptake and plant growth in low Pi environments.

## Teaser

Overexpression of a chloroplastic protein involved in lipid metabolism improves phosphate uptake and growth in low-phosphate environments in Arabidopsis and rice.

## Introduction

Phosphorus (P) is a macronutrient and an essential constituent of numerous organic compounds required for plant growth and development, such as nucleic acids, nucleoside triphosphates, sugar phosphates, phosphoproteins, and phospholipids. Thus, to synthesize these P-containing organic compounds, plants require a large amount of P, which is acquired from the rhizosphere as phosphate (Pi), mostly in the form of dihydrogen phosphate (H_2_PO_4_^−^) and hydrogen phosphate (HPO_4_^2−^). However, Pi in soils readily reacts with cations, forming insoluble compounds (Schachtman et al. 1998). Hence, despite the high content of total P in the soil, plants often encounter a severe deficiency of Pi in the natural ecosystem and the field (Abel et al. 2002).

When exposed to Pi deficiency stress, plants exhibit appropriate responses, which include improving the efficiency of Pi uptake from the rhizosphere and that of Pi utilization in the plant body. To enhance Pi uptake efficiency, plants promote the synthesis of organic acids, such as citrate, malate, and oxalate, and secrete them into the rhizosphere to release Pi from insoluble P-containing compounds (Raghothama and Karthikeyan 2005). Plants also secrete acid phosphatases for the hydrolytic release of Pi from Pi monoesters in the rhizosphere (Plaxton 2004). Furthermore, plants activate the expression of Pi acquisition-related genes. Members of the PHOSPHATE TRANSPORTER 1 (PHT1) family, a group of Pi transporters mainly localized in the plasma membrane (Nussaume et al. 2011), play roles in Pi uptake from the rhizosphere and in the xylem loading of Pi for root-to-shoot transfer. By contrast, members of other Pi transporter families, such as PHT2-5 in *Arabidopsis* (*Arabidopsis thaliana*) and PHT2-4 in rice (*Oryza sativa* L.), play roles in intracellular Pi transport, according to their plastid, mitochondria, or tonoplast localization. Therefore, Pi deficiency stress induces *PHT1* genes as well as other genes and microRNAs (miRNAs) critical for Pi deficiency responses. The activation of gene expression by Pi deficiency involves PHOSPHATE STARVATION RESPONSE 1 (PHR1) and its functional homologs, and SYG1/Pho81/XPR1 (SPX) proteins (Puga et al. 2017; Wang et al. 2018). The PHR1 family proteins are transcriptional activators that directly activate Pi starvation-inducible genes, while SPX proteins interact with PHR1 family proteins and suppress their activity when Pi is sufficient (Puga et al. 2017; Wang et al. 2018). Furthermore, miR399 decreases the transcript level of *PHO2*, which encodes a ubiquitin-conjugating E2 enzyme involved in the ubiquitination and degradation of PHT proteins (Fujii et al. 2005; Bari et al. 2006; Chiou et al. 2006). Thus, Pi uptake is regulated by a combination of transcriptional and posttranscriptional controls, depending on Pi availability.

To utilize endogenous Pi more efficiently in response to Pi deficiency, plants reallocate Pi pools to maintain adequate levels of nucleic acids, ATP, etc. In membrane lipid remodeling under Pi deficiency, plants replace phospholipids in membranes with P-free lipids (galactolipids and sulfolipids) and hydrolyze the phospholipids to release Pi. Because 2 uncharged galactolipids, monogalactosyldiacylglycerol (MGDG) and digalactosyldiacylglycerol (DGDG), are the primary components of the plastid envelope and thylakoid membranes (Hölzl and Dörmann 2019), chloroplast membranes are less dependent on phospholipids, unlike other biomembranes. However, chloroplast membranes also contain phosphatidylglycerol (PG), a phospholipid with a negative charge, which is necessary for the negative charge on thylakoid membranes and accounts for 5% to 15% of chloroplast lipids (Hölzl and Dörmann 2019). Therefore, under Pi-deficient conditions, plants decrease the accumulation of PG, promote the synthesis of sulfoquinovosyldiacylglycerol (SQDG; an anionic glycolipid), and replace PG with SQDG (Yu et al. 2002), maintaining the abundance of anionic lipids in the thylakoid membrane.

The Sec14 protein was identified in yeast as a lipid transfer protein, mediating the transport of phosphatidylinositol (PI) and phosphatidylcholine (PC) between membrane bilayers (Szolderits et al. 1989). Since then, Sec14-like proteins, defined as PI transfer proteins (PITPs), have been reported in various eukaryotic unicellular and multicellular organisms but not in prokaryotic bacteria (Allen-Baume et al. 2002). Vascular plants contain ∼30 Sec14-like proteins. For example, *Arabidopsis* and rice possess 32 Sec14-like proteins (AtPITP1-32) and 29 Sec14-like proteins (OsPITP1-29), respectively (Huang et al. 2016). Plant Sec14-like proteins have a much more complex structure than the yeast ones because of the presence of additional functional domains, including the nodulin and GOLD domains. Furthermore, plant Sec14-like proteins are likely to present different subcellular localization patterns due to their diverse functions (Huang et al. 2016). Although the functions of most plant Sec14-like proteins remain unknown, some of these proteins have been shown to perform distinct functions. For instance, *Arabidopsis* COW1, a characterized plant Sec14-like protein, possesses a nodulin domain and plays a role in the promotion of root hair tip growth (Böhme et al. 2004). Another plant Sec14-like protein, PATL1 with a GOLD domain, localizes to the cell plate in roots, specifically binds to several phosphoinositides, including phosphatidylinositol 5-phosphate (PI5P) and phosphatidylinositol 4,5-bisphosphate (PI(4,5)P_2_), and acts as a regulator of membrane trafficking in *Arabidopsis* (Peterman et al. 2004). Furthermore, a recent study revealed that *Arabidopsis* PITP7 (designated as CHLOROPLAST-LOCALIZED SEC14-LIKE PROTEIN [CPSFL1]) localizes to chloroplasts, binds to phosphatidic acid (PA) and phosphatidylinositol 4-phosphate (PI4P) in vitro, and plays an essential role in photoautotrophic growth and chloroplast development (Hertle et al. 2020).

In the current study, we found that the function of *AtPITP7* was strongly associated with differences in Pi uptake activity among *Arabidopsis* accessions. Further investigation of AtPITP7 and its rice homolog OsPITP6 revealed that both AtPITP7 and OsPITP6 are chloroplastic Sec14-like proteins involved in Pi acquisition and lipid remodeling. Based on the phenotype of transgenic rice lines overexpressing *OsPITP6*, we also suggest that OsPITP6 and its homologs in other crops may be useful for developing crops with enhanced Pi uptake activity and improved growth under Pi-limiting conditions.

## Results

### AtPITP7 is involved in Pi uptake

**Figure 1.**
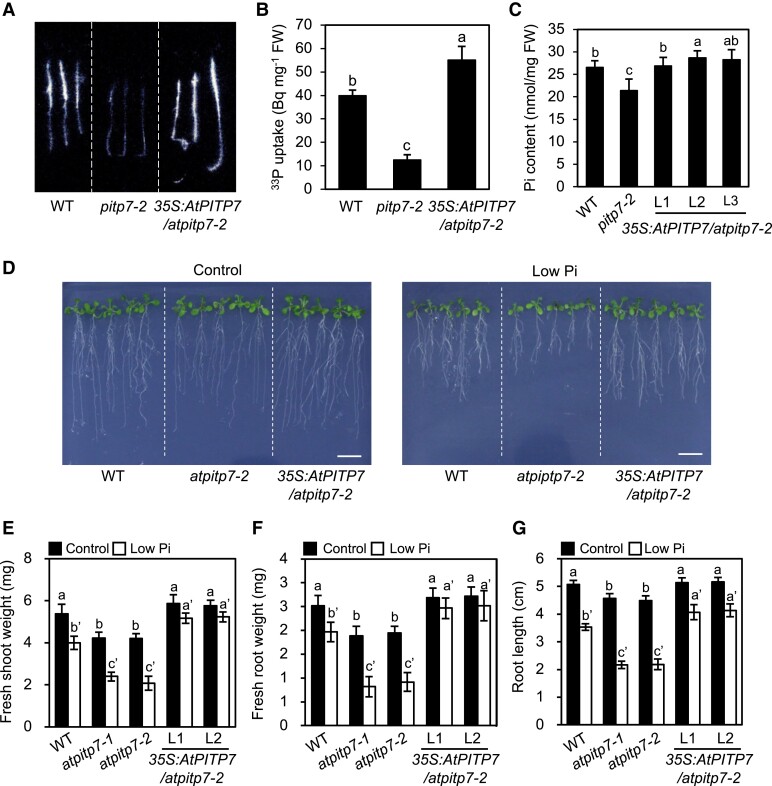
Effects of the knockout mutation and overexpression of *AtPITP7* on Pi uptake and growth of *Arabidopsis* seedlings under Pi-deficient conditions. **A)** Visualization and **B)** quantification of ^33^P-labeled P_i_ uptake by 10-d-old WT, *atpitp7-2*, and *35S:AtPITP7/atpitp7-2*/(L1) seedlings grown on 1/2 MS agar plates for 5 d and then subjected to Pi deficiency treatment for 5 d. **C)** Quantification of Pi in 12-d-old WT, *atpitp7-2*, and *35S:AtPITP7/atpitp7-2* (L1 to L3) seedlings grown on 1/2 MS agar plates. **D)** Phenotype, **E)** fresh shoot weight, **F)** fresh root weight, and **G)** root length of 15-d-old WT, *atpitp7-2*, and *35S:AtPITP7/atpitp7-2* (L1) seedlings grown on 1/2 MS agar plates for 5 d and then on control Pi (500 *μ*M Pi) or low Pi (5 *μ*M Pi) agar plates for 10 d. In **B)**, **C)**, and **E** to **G)**, data represent mean ± standard deviation (Sd) of 5 biological replicates, and different letters above bars indicate statistically significant differences (*P* < 0.05; Tukey's multiple comparison test). Scale bar = 1 cm **D)**.

In the genome-wide association study (GWAS) of 133 *Arabidopsis* accessions showing differences in Pi uptake activity under Pi-deficient conditions (Sakuraba et al. 2018), single nucleotide polymorphisms (SNPs) with small *P*-values were found most frequently in the 5′-flanking region of *AtPITP7* (At5g63060) on chromosome 5, suggesting an association of *AtPITP7* with Pi uptake (Supplemental Fig. S1). We investigated further using 2 *Arabidopsis* T-DNA insertion lines in the Col-0 background, SALK_047586C (*atpitp7-1*) and SALK_116713C (*atpitp7-2*). Both *atpitp7-1* and *atpitp7-2* are knockout mutants since they carry T-DNA insertions in the 1st and 4th exons, respectively, and did not produce *AtPITP7* transcripts (Supplemental Fig. S2). We also employed transgenic *Arabidopsis* plants harboring the *35S:AtPITP7-MYC* cassette in the *atpitp7-2* mutant background (*35S:AtPITP7/atpitp7-2*) (Supplemental Fig. S3A). Transcript levels of *AtPITP7-MYC* in 3 independent *35S:AtPITP7/atpitp7-2* lines (L1 to L3) were >6-fold higher than that of the endogenous *AtPITP7* gene in wild-type (WT; Col-0) plants (Supplemental Fig. S3B). ^33^P-Labeled Pi uptake assay revealed that, compared with WT seedlings, Pi uptake activity was lower in *atpitp7-1* and *atpitp7-2* seedlings but higher in *35S:AtPITP7/atpitp7-2* seedlings (Figs. 1, A and B, and S4). The seedling Pi content showed a similar trend, although the increase in Pi content of *335S:AtPITP7/atpitp7-2* seedlings relative to the WT was not significant (Figs. 1, C, and S4). Furthermore, both *atpitp7-1* and *atpitp7-2* mutants exhibited growth defects, lower fresh shoot and root weights, and shorter primary roots compared with the WT under both Pi-sufficient (control) and low Pi conditions, although these phenotypes were more enhanced under low Pi conditions (Fig. 1, D to G). Note that the growth defects of *atpitp7* mutants were milder under the current continuous light condition than under the 12 h light/12 h dark condition where the severe growth defects of *atpitp7* mutants were previously observed (Hertle et al. 2020) (Supplemental Fig. S5). Compared with *atpitp7* mutants, plants of *35S:AtPITP7/atpitp7-2* lines L1 and L2 exhibited opposite phenotypes under low Pi conditions (Fig. 1, D to G). These results suggest that the *AtPITP7* expression level is associated with Pi uptake activity and Pi availability-dependent growth modulation.

### Association between natural variation in Pi uptake activity and *AtPITP7* promoter activity

**Figure 2.**
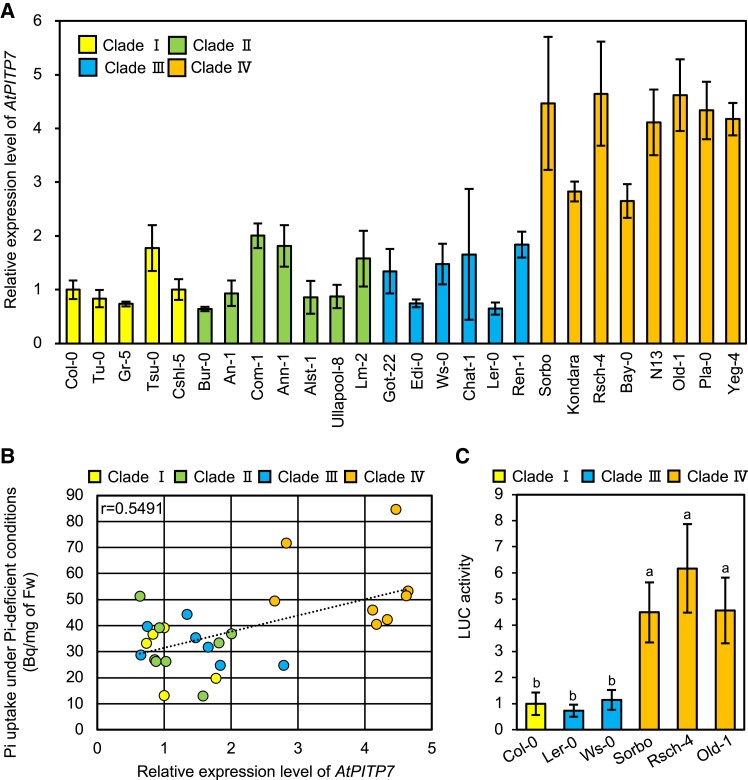
Natural variation in *AtPITP7* expression among 26 *Arabidopsis* accessions. **A)** Expression levels of *AtPITP7* in 8-d-old *Arabidopsis* seedlings. Transcript levels of *AtPITP7* were normalized against that of *ACT2*. **B)** Correlation between *AtPITP7* expression level and Pi uptake activity. **C)** Relative LUC activity derived from the expression of *LUC* reporter gene under the control of Col-0-, Ler-0-, Ws-0-, Old-1-, Sorbo-, or Rsch-4-type *AtPITP7* promoter in *Arabidopsis* protoplasts. In **A**, **C)**, data represent mean ± Sd of 4 biological replicates, and different letters above bars indicate statistically significant differences (*P* < 0.05; Tukey's multiple comparison test).

Consistent with the chloroplast localization of AtPITP7 (Hertle et al. 2020), *AtPITP7* transcripts were much more abundant in shoots than in roots (Supplemental Fig. S6). Furthermore, the *AtPITP7* expression level in shoots remarkably increased during Pi deficiency (Supplemental Fig. S6), further supporting the association of the *AtPITP7* expression level with Pi deficiency responses. This association was first recognized through the GWAS (Supplemental Fig. S1) and then confirmed by subsequent phenotypic analyses (Fig. 1). Therefore, we next investigated the association between the *AtPITP7* expression level and Pi uptake activity using a collection of diverse *Arabidopsis* accessions. The *AtPITP7* promoter was previously sequenced in 102 out of 135 accessions used in the GWAS (Supplemental Table S1). Phylogenetic analysis of these *AtPITP7* promoter sequences (downloaded from the 1001 Genomes database; http://signal.salk.edu/atg1001/3.0/gebrowser.php) revealed 4 clades (I to IV) (Supplemental Fig. S7). Analysis of *AtPITP7* expression levels in randomly selected accessions revealed that the *AtPITP7* promoters in clade IV directed higher gene expression levels than those in the other clades (Fig. 2, A). Furthermore, the scatter plot presenting the relationship between the expression level of *AtPITP7* and Pi uptake activity revealed a close association between the 2 (Fig. 2, B).

Amino acid sequence alignment of AtPITP7 proteins from 5 accessions belonging to different clades revealed mostly conserved sequences, except for 2 amino acid substitutions in the chloroplast-targeting sequence (Supplemental Fig. S8). Similarly, no or few polymorphisms were found among the nucleotide sequences of *AtPITP7* promoters belonging to clades I to III (Supplemental Fig. S9). However, many polymorphisms were detected in the clade IV *AtPITP7* promoters (Supplemental Fig. S10). Several polymorphisms were found to be highly conserved in the accessions of clade IV, although most of the polymorphisms identified were not associated with known *cis*-elements (Supplemental Fig. S10). Protoplast transient assays performed using the *AtPITP7* promoters of 6 accessions revealed that *AtPITP7* promoters of clade IV were remarkably stronger than those of other clades (Fig. 2, C), suggesting that some of the nucleotide polymorphisms found in clade IV *AtPITP7* promoters are responsible for their more potent activity, leading to higher *AtPITP7* expression levels and Pi uptake activity in the corresponding accessions.

### Identification of the rice homolog of AtPITP7

Enhanced Pi uptake is an agronomically superior trait. To investigate whether overexpression of the rice homolog of *AtPITP7* would enhance Pi uptake in rice, as was the case for overexpression of *AtPITP7* in *Arabidopsis*, we first identified the homolog in rice. In *Arabidopsis*, of the 32 Sec14-like proteins, only AtPITP7 has been predicted to localize to chloroplasts as it contains a chloroplast-targeting sequence (Supplemental Fig. S11). Therefore, we searched for proteins containing a chloroplast-targeting sequence and showing sequence similarity to AtPITP7 using the genome sequences of rice and other 5 plant species, including poplar (*Populus trichocarpa*), oilseed rape (*Brassica napus*), tomato (*Solanum lycopersicum*), tobacco (*Nicotiana tabacum*), and maize (*Zea mays*). Each plant species possessed only 1 gene satisfying these criteria (Supplemental Fig. S12), and the candidate for the rice homolog of *AtPITP7* was Os02g0321500 (designated as *OsPITP6*) (Supplemental Fig. S13). Like *AtPITP7*, *OsPITP6* was expressed to higher levels in shoots than in roots and was induced by Pi deficiency treatment (Supplemental Fig. S14). Furthermore, OsPITP6-green fluorescent protein (GFP) fusion protein was localized to chloroplasts when transiently expressed in rice protoplasts (Supplemental Fig. S15A). Immunoblot analysis using chloroplast subfractions prepared from transgenic rice plants expressing the *OsPITP6*-*MYC* chimeric gene (details are described in the next section) revealed that OsPITP6-MYC localized to the chloroplast stroma and not to the thylakoid membrane or chloroplast envelope (Supplemental Fig. S15B). Hence, OsPITP6 was confirmed as the rice homolog of AtPITP7.

### Effects of *OsPITP6* overexpression and inactivation on Pi uptake and plant growth

To investigate whether, like AtPITP7, OsPITP6 is involved in the regulation of Pi uptake, we first inactivated *OsPITP6* in the rice cultivar Nipponbare using the CRISPR/Cas9 technology. Then, we established 3 independent mutant lines (referred to as *ospitp6-1*, *-2*, and *-3*) from 3 independent T0 plants carrying biallelic mutations in *OsPITP6*, which produced short OsPITP6 proteins (Supplemental Fig. S16). We also overexpressed the *OsPITP6-MYC* chimeric gene under the control of the maize *Ubiquitin* promoter to generate transgenic rice lines (referred to as *Ubi:OsPITP6*) (Supplemental Fig. S17A). Transcript levels of *OsPITP6-MYC* in 8 independent lines were >10-fold higher than that of *OsPITP6* in the WT (Supplemental Fig. S17B). Two independent *Ubi:OsPITP6* lines (L6 and L8) were randomly selected and used for further analysis.

**Figure 3.**
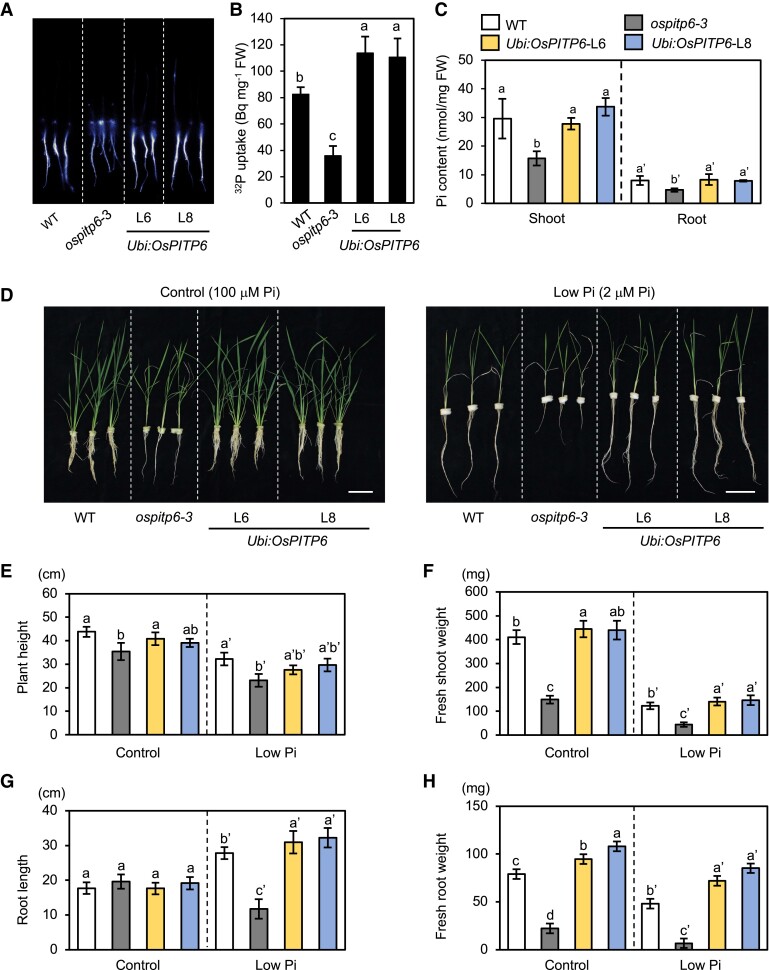
Pi uptake and Pi deficiency responses of *ospitp6*-*3* mutant and *OsPITP6* overexpression (*Ubi:OsPITP6*) rice lines. **A)** Image and **B)** quantification of ^32^P-labeled Pi uptake in WT, *ospitp6*-*3*, and *UBI:OsPITP6* seedlings grown in 0.5× Yoshida nutrient solution for 7 d (after germination) and then in low Pi nutrient solution for 5 d. **C)** Pi concentration in shoots and roots of WT, *ospitp6*-3, and *Ubi:OsPITP6* seedlings grown in 0.5× Yoshida nutrient solution for 7 d (after germination) and then in the control Pi nutrient solution for 5 d. **D)** Phenotype, **E)** height, **F)** fresh shoot weight, **G)** root length, and **H)** fresh root weight of WT, *ospitp6*-*3*, and *Ubi:OsPITP6* seedlings grown in 0.5× Yoshida solution for 7 d and then in the control Pi (100 *µ*M) nutrient solution for 10 d. The seedlings were finally grown in the control Pi or low Pi (2 *µ*M) nutrient solution for 25 d. Scale bar = 10 cm. In **B)**, **C)**, and **E** to **H)**, data represent mean ± Sd of 4 **C)** or 5 **B)** and **E** to **H)** biological replicates, and different letters above bars indicate statistically significant differences (*P* < 0.05; Tukey's multiple comparison test).

The effects of *OsPITP6* inactivation and overexpression on Pi uptake activity were examined by exposing young seedlings to Pi deficiency. Compared with the WT, Pi uptake activity was lower in *ospitp6*-*3* seedlings but higher in *Ubi:OsPITP6* seedlings (Fig. 3, A and B). Furthermore, Pi contents of both shoots and roots were lower in *ospitp6-3* seedlings than in the WT, although no significant difference was detected between the WT and *Ubi:OsPITP6* seedlings, suggesting that increases in Pi taken up in *Ubi:OsPITP6* plants were mostly consumed for growth promotion (Fig. 3, C). Consistent with this idea, *OsPITP6* inactivation also affected the P content, although its effects on carbon (C) and nitrogen (N) contents were minimal (Supplemental Fig. S18). Next, we characterized the phenotype of WT, *ospitp6-3*, and *Ubi:OsPITP6* (L6 and L8) seedlings grown hydroponically under control and low Pi conditions. The *ospitp6-3* seedlings were short and exhibited decreased fresh shoot and root weights under both conditions and displayed a reduction in root length under low Pi conditions (Fig. 3, D to H); similar growth defects were observed in *ospitp6-1* and *ospitp6-2* mutants (Supplemental Fig. S19). On the other hand, shoot growth of *Ubi:OsPITP6* seedlings was mostly comparable with that of WT seedlings, while root growth was better in *Ubi:OsPITP6* seedlings than in the WT, especially when grown in the low Pi solution (Fig. 3, D to H). Furthermore, relative C/N, C/P, and N/P ratios of WT and *ospitp6-3* seedlings, which were calculated with C, N, and P contents in Supplemental Fig. S18, indicated that P deficiency exerted a similar and limited effect on the C/N ratio in WT and *ospitp6-3* seedlings but markedly increased C/P and N/P ratios in both shoots and roots of WT and *ospitp6-3* seedlings (Supplemental Table S2). Furthermore, the inactivation of *OsPITP6* enhanced P deficiency-induced increases in C/P and N/P ratios. These results suggest that the role of *OsPITP6* is more closely related to the acquisition of P than C and N.

Considering that *ospitp6* plants showed impaired growth even under the Pi-sufficient condition, OsPITP6 and AtPITP7 are probably involved in photoautotrophic growth. On the other hand, these results also suggest that OsPITP6, like AtPITP7, also participates in the modulations of Pi uptake activity and Pi deficiency responses.

### Inactivation of *OsPITP6* accelerates singlet oxygen generation in leaves

**Figure 4.**
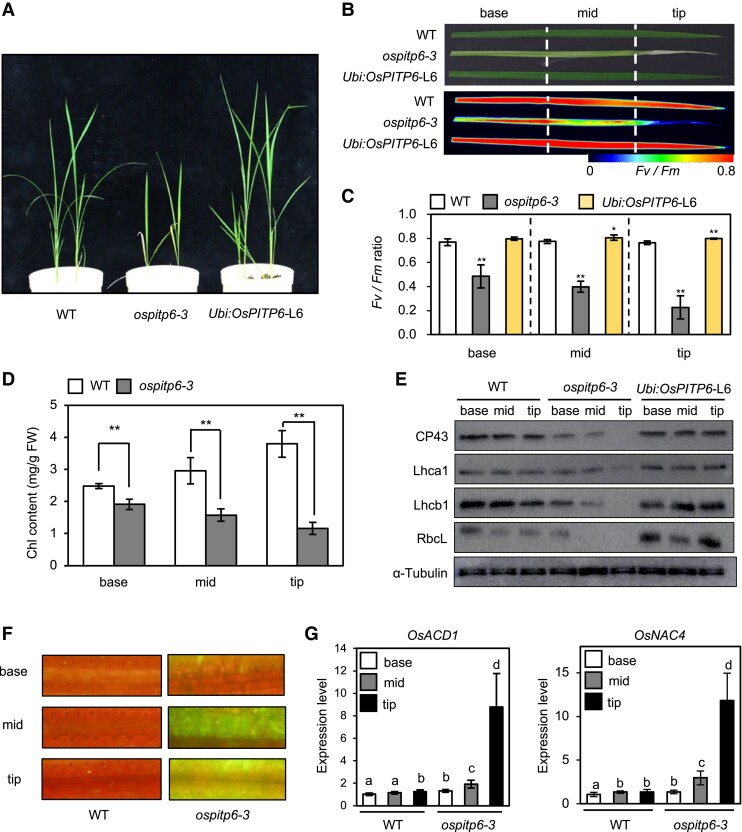
Characterization of the necrotic phenotype of *ospitp6-3* leaves. **A)** Photographs of 3-w-old WT, *ospitp6*-*3*, and *Ubi:OsPITP6* (L6) seedlings grown in soil in the greenhouse under long-day conditions (16 h light/8 h dark) with a halide lamp (light intensity: 1,100 *μ*mol m^−2^ s^−1^). **B)** Leaf color and *Fv/Fm* image, **C)***Fv/Fm* ratio, **D)** total chlorophyll (Chl) content, and **E)** levels of photosynthesis-associated proteins (CP43, Lhcb1, Lhca1, RbcL, and α-tubulin) in the base, middle (mid), and tip regions of leaf blades of 45-d-old WT, *ospitp6*-*3*, and *Ubi:OsPITP6* (L6) plants grown in the soil. **F)** Singlet Oxygen Sensor Green (SOSG) fluorescence image showing singlet oxygen production in the base, middle (mid), and tip regions of the leaf blades of 45-d-old WT and *ospitp6*-*3* rice plants. **G)** RT-qPCR analysis of cell-death marker genes (*OsACD1* and *OsNAC4*) in the base, middle (mid), and tip regions of the leaf blades of 45-d-old WT and *ospitp6*-*3* plants. Gene transcript levels were normalized first against the transcript level of *OsUBQ5* and then against the value obtained from the base of WT leaf blades. Data represent the mean ± Sd of 5 **C)** or 4 **D)** biological replicates. In **C**, **D)**, asterisks indicate significant differences between WT and *ospitp6-3* samples (*P < 0.05, **P < 0.01; Student's *t*-test). In **G)**, different letters above bars indicate statistically significant differences (*P* < 0.05; Tukey's multiple comparison test).

Consistent with the potential role of OsPITP6 in photoautotrophic growth, soil-grown *ospitp6-3* plants exhibited a semidwarf phenotype (Fig. 4, A) and necrosis of leaf tips (Fig. 4, A and B). Furthermore, the maximum quantum yield of photosystem II (*Fv/Fm*), chlorophyll content, and photosystem protein contents were much lower in *ospitp6*-*3* leaves than in WT and *Ubi:OsPITP6* leaves, indicating reduced photosynthetic activity in *ospitp6-3* leaves (Fig. 4, C to E). Furthermore, the generation of singlet oxygen, 1 of the reactive oxygen species (ROS) that induces cell death (Apel and Hirt 2004), and the expression of 2 cell-death associated genes, *OsACD1* and *OsNAC4* (Kaneda et al. 2009; Tang et al. 2011), were promoted in *ospitp6-3* leaves (Fig. 4, F and G). These results suggest that OsPITP6 is required for the maintenance of photosynthetic activity in chloroplasts.

### Opposite effects of *OsPITP6* inactivation and overexpression on rice grain yield

**Figure 5.**
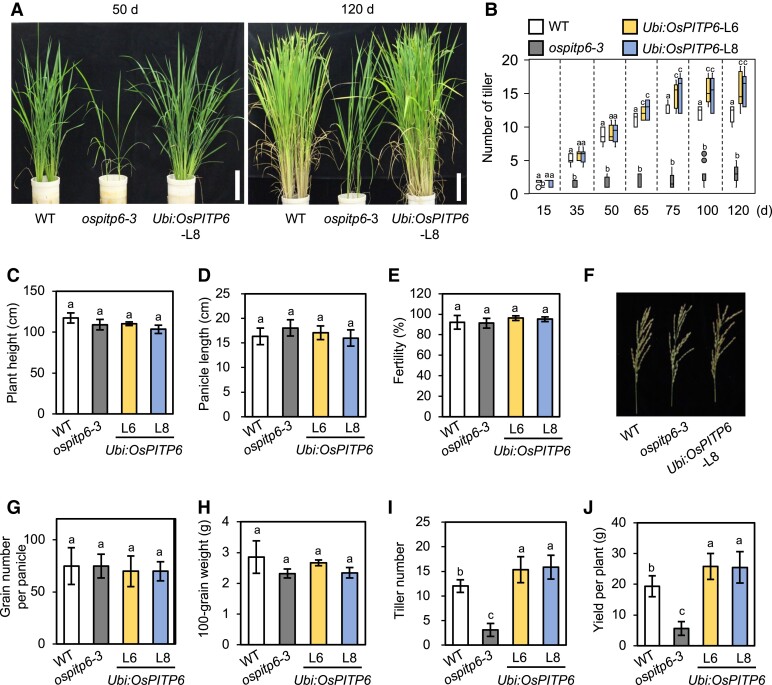
Agronomic traits of *ospitp6-3* and *UBI:OsPITP6* rice plants. **A)** Photographs of 50-d-, and 120-d-old WT, *ospitp6-3*, and *Ubi:OsPITP6* (L8) rice plants grown in soil. **B)** Tiller number of 15-d-, 35-d-, 50-d-, 65-d-, 75-d-, 100-d-, and 120-d-old soil-grown WT, *ospitp6-3*, and *Ubi:OsPITP6* (L6 and L8) plants. Horizontal lines in the box denote median values. Boxes extend from the 25th percentile to the 75th percentile of each line's distribution of values. Dots denote observations outside the range of adjacent values. **C)** Height, **D)** panicle length, **E)** seed fertility, **F)** panicle phenotype, and **G)** grain number per **H)** panicle 100-grain weight, **I)** tiller number, **J)** and yield per plant of 150-d-old WT, *ospitp6-3*, and *Ubi:OsPITP6* (L6 and L8) plants grown in soil. In **B)**, **C** to **E)**, and **G** to **J)**, data represent mean ± Sd of 5 biological replicates, and different letters above bars indicate statistically significant differences (*P* < 0.05; Tukey's multiple comparison test).

The *ospitp6-3* and *Ubi:OsPITP6* plants were grown in soil until the reproductive stage, and the positive and negative effects of *OsPITP6* inactivation and overexpression were observed at all growth stages (Fig. 5, A and B). The final height of WT, *ospitp6-3*, and *Ubi:OsPITP6* plants showed no significant differences at the reproductive stage (after 150 d of growth) (Fig. 5, C). Furthermore, genetic modifications of *OsPITP6* did not affect the grain number per panicle, seed fertility percentage, and 100-grain weight but affected the tiller number and grain yield per plant (Fig. 5, D to J). Interestingly, the effect of *OsPITP6* overexpression on tiller number was not pronounced at the early vegetative growth stage but was evident at the late vegetative growth stage (Figs. 5, B, and S20). Since the *OsPITP6-MYC* transcript level in *Ubi:OsPITP6* was maintained at a constant level from the early seedling stage to the heading stage and always much higher than the *OsPITP6* transcript level in the WT (Supplemental Fig. S21), it is probable that the positive effects of *OsPITP6* overexpression accumulated over time and then led to increased grain yield per plant by promoting tilling. Importantly, *OsPITP6* overexpression increased the tiller number even when seedlings were grown in the low Pi solution, and the positive effects appeared to be stronger under low Pi conditions than under control conditions (Supplemental Fig. S22), consistent with the growth of *Ubi:OsPITP6* plants (Fig. 3).

### Altered lipid profiles of *ospitp6-3* and *Ubi:OsPITP6* plants

AtPITP7 has been identified as a chloroplast protein capable of binding to phospholipids, such as PI and PA, in vitro (Hertle et al. 2020), and Pi deficiency reduces phospholipid contents but increases glycolipid contents (Okazaki et al. 2013). To access the molecular basis of the superior phenotype of *Ubi:OsPITP6* plants, we investigated the contents of 4 phospholipids (PI, PA, PG, and PC) and 3 glycolipids (MGDG, DGDG, and SQDG) in the leaves and purified chloroplasts of WT, *ospitp6-3*, and *Ubi:OsPITP6* seedlings.

**Figure 6.**
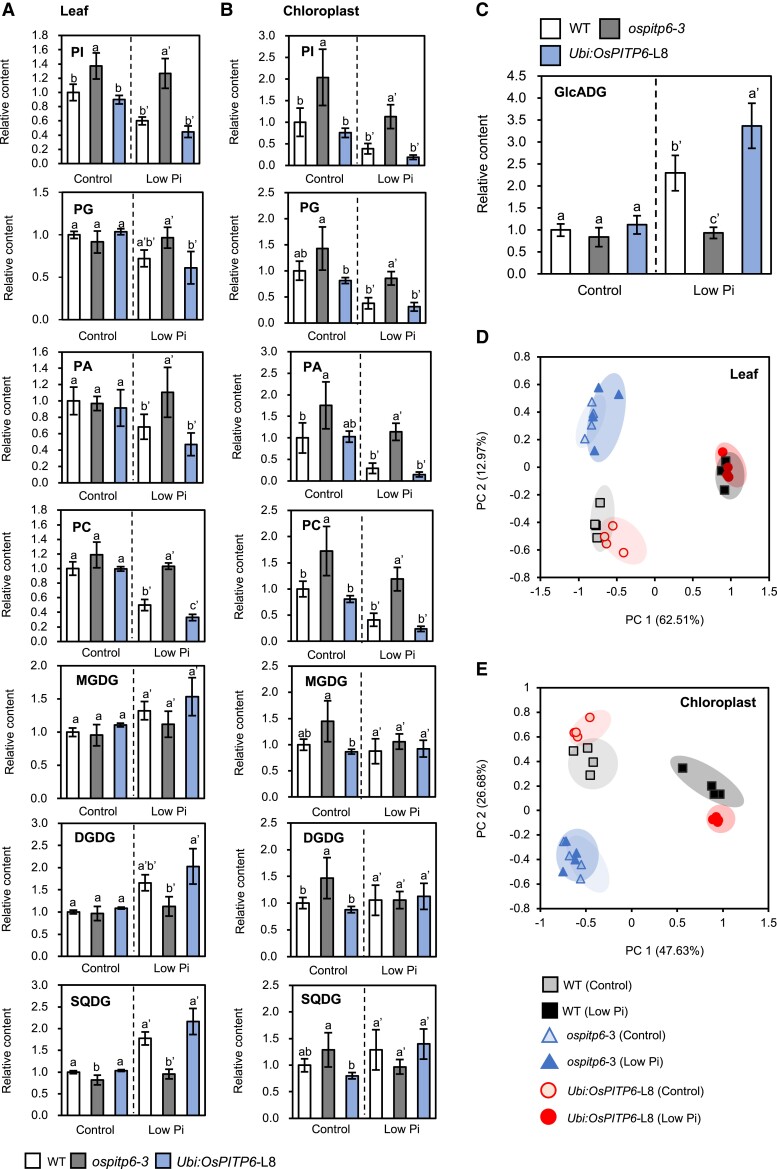
Phospholipid and glycolipid contents of the leaves and chloroplasts of WT, *ospitp6-3*, and *UBI:OsPITP6* plants. **A**, **B)** Relative phospholipid and glycolipid contents of **A)** leaves and **B)** chloroplasts of 4-w-old WT, *ospitp6-3*, and *Ubi:OsPITP6* (L8) seedlings that were grown in 0.5× Yoshida nutrient solution for 2 w and then in the control or low Pi nutrient solution for 2 w. Values obtained in *ospitp6-3* and *Ubi:OsPITP6* (L8) seedlings were normalized relative to those obtained in WT seedlings grown in the control nutrient solution. PI, phosphatidylinositol; PA, phosphatidic acid; PG, phosphatidylglycerol; PC, phosphatidylcholine; MGDG, monogalactosyldiacylglycerol; DGDG, digalactosyldiacylglycerol; SQDG, sulfoquinovosyldiacylglycerol. **C)** Glucuronosyl diacylglycerol (GlcADG) content in whole leaf samples. In **A)** to **C)**, data represent mean ± Sd of 5 biological replicates, and different letters above bars indicate statistically significant differences (*P* < 0.05; Tukey's multiple comparison test). Principal component analysis (PCA) of PI, PA, PG, PC, MGDG, DGDG, and SQDG in **D)** leaves and **E)** chloroplasts of 4-w-old WT, *ospitp6-3*, and *Ubi:OsPITP6* (L8) seedlings. PC1 and PC2 explained 64.32% and 13.05% of the variation, respectively, in **D)** and 50.33% and 16.63% of the variation, respectively, in **E)**.

The phospholipid and glycolipid profiles were similar between WT and *Ubi:OsPITP6* plants in both leaf and chloroplast samples, regardless of the Pi concentration in the nutrient solution. However, Pi deficiency-dependent changes in lipid profiles were enhanced in *Ubi:OsPITP6* plants (Fig. 6, A and B). For instance, PC content showed no significant differences between WT and *Ubi:OsPITP6* plants grown in the control Pi solution; however, the PC content was lower in *Ubi:OsPITP6* plants than in the WT when grown in low Pi conditions. Conversely, the content of glucuronosyl diacylglycerol (GlcADG) in the leaves, which was additionally measured because the GlcADG content increases under Pi deficiency stress in *Arabidopsis* (Okazaki et al. 2013), was more significantly increased by Pi deficiency stress in *Ubi:OsPITP6* leaves than in WT leaves (Fig. 6, C).

Unlike the case of *Ubi:OsPITP6* plants, the lipid profiles of WT and *ospitp6*-*3* plants were different even when grown in the control Pi solution. In seedlings grown under Pi-sufficient conditions, the differences between chloroplast samples were more evident than those between leaf samples, probably because leaf and chloroplast sample values were normalized relative to the leaf fresh weight and chlorophyll content, respectively. In fact, MGDG and DGDG, which are mostly present in chloroplast membranes (Hölzl and Dörmann 2019), were similar in content between *ospitp6-3* and WT leaf samples. Thus, when Pi was sufficient, the inactivation of *OsPITP6* likely affected the lipid profile slightly but chlorophyll accumulation more prominently. On the other hand, under Pi-deficient conditions, Pi deficiency-induced changes in phospholipid and glycolipid contents were alleviated in *ospitp6-3* seedlings, even though these seedlings were likely more severely deficient in Pi, because of the lower Pi uptake activity and Pi content than in WT seedlings (Fig. 3).

Principal component analysis (PCA), conducted with PI, PA, PG, PC, MGDG, DGDG, and SQDG contents to understand the genotype- and Pi condition-dependent alterations in phospholipid and glycolipid contents, fully supported our conclusion (Fig. 6, D and E). Two profiles observed under control and low Pi conditions were evidently separated in samples from WT and *Ubi:OsPITP6* plants but not in samples from *ospitp6-3* plants. Furthermore, although it was difficult to distinguish between the lipid profiles of WT and *Ubi:OsPITP6* seedlings under control Pi conditions, they could be distinguished under low Pi conditions. Therefore, we concluded that OsPITP6 plays an essential role in balancing phospholipid and glycolipid syntheses in chloroplasts in response to Pi availability, and overexpression of *OsPITP6* enhances Pi deficiency responses in chloroplasts.

### Modified expression levels of Pi uptake-related genes in *ospitp6-3* plants

**Figure 7.**
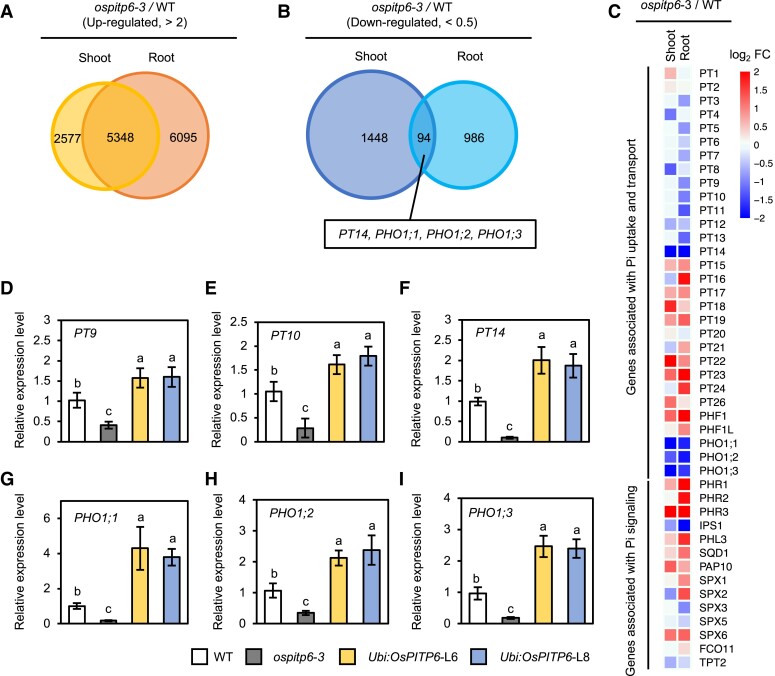
Antagonistic effects of *OsPITP6* overexpression and inactivation on Pi-related gene expression. Venn diagrams representing numbers of **A)** upregulated (FC > 2) genes and **B)** downregulated (FC < 0.5) genes in the DNA microarray analysis of shoots and roots of WT and *ospitp6*-*3* plants grown first in 0.5× Yoshida solution for 7 d and then grown in soil for 3 w. Pi-responsive genes downregulated in both the shoots and roots of *ospitp6*-*3* plants are indicated. **C)***ospitp6*-*3* allele-dependent modulations in the expression levels of Pi uptake-, translocation-, and signaling-related genes. RT-qPCR analysis of the expression levels of **D)***PT9*, **E)***PT10*, **F)***PT14*, **G)***PHO1;1*, **H)***PHO1;2*, and **I)***PHO1;3* in the roots of WT, *ospitp6-3*, and *Ubi:OsPITP6* (L6 and L8) plants grown in 0.5× Yoshida solution for 7 d and then in soil for 3 w. Data represent mean ± Sd of 5 biological replicates, and different letters above bars indicate statistically significant differences (*P* < 0.05; Tukey's multiple comparison test).

To gain further insight into the role of OsPITP6 in regulating Pi uptake activity and Pi deficiency responses, we compared the shoot and root transcriptomes of WT and *ospitp6*-*3* seedlings grown under control Pi conditions. Genes upregulated (>2-fold, *P* < 0.05) in *ospitp6-3* plants (7,925 in shoots and 11,443 in roots) were substantially more than those downregulated (<0.5-fold, *P* < 0.05) (1,542 in shoots and 1,080 in roots), revealing the pleiotropic effects of the inactivation of *OsPITP6* (Fig. 7, A and B). Hierarchical average linkage cluster analysis revealed that the inactivation of *OsPITP6* affected the expression levels of different sets of genes in shoots and roots (Supplemental Fig. S23), suggesting that the expression level of *OsPITP6* influences different sets of physiological processes in shoot and root tissues. Although the highly upregulated or downregulated genes did not include Pi-related genes (Supplemental Tables S3 to S6), a detailed analysis of the expression levels of Pi-related genes in roots revealed that *OsPT1* to *OsPT13* genes (encoding PHT1-class Pi transporters, mainly involved in Pi uptake in roots) were downregulated only slightly in *ospitp6-3* seedlings. Interestingly, among Pi-related genes, *OsPT14*, which encodes a PHT2-class Pi transporter that mediates Pi import into chloroplasts (Shi et al. 2013), and 3 *PHOSPHATE1* (*OsPHO1*) genes involved in Pi translocation (Chiou 2020) showed the most remarkable downregulation in both shoots and roots of *ospitp6-3* plants (Fig. 7, C; Supplemental Tables S7, S8). Furthermore, reverse transcription quantitative PCR (RT-qPCR) analysis revealed significant opposite effects of *OsPITP6* inactivation and overexpression on the expression levels of 6 Pi uptake- and transport-related genes (*OsPT9*, *OsPT10*, *OsPT14*, *OsPHO1;1*, *OsPHO1;2*, and *OsPHO1;3*) (Fig. 7, D to I).

### Chloroplast AtPIP7 is required for proper regulation of Pi uptake and plant growth in *Arabidopsis*

**Figure 8.**
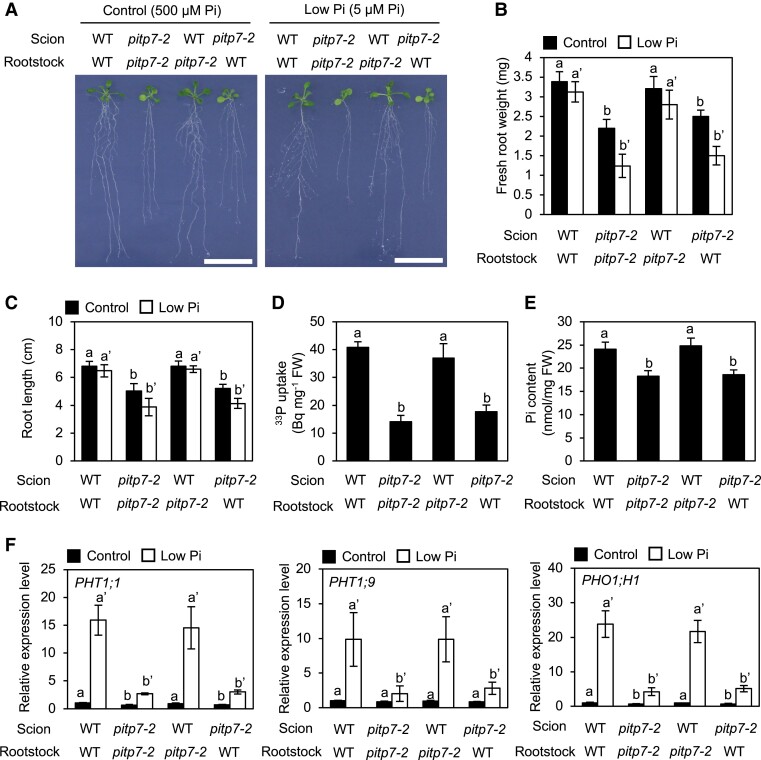
*AtPITP7* inactivation in shoots causes root growth impairment and reduced Pi uptake in roots. **A)** Phenotype, **B)** fresh root weight, **C)** root length, **D)**^33^P-labeled Pi uptake, and **E)** Pi content of grafted *Arabidopsis* seedlings generated using WT and *atpitp7*-2 scions and rootstocks. Grafted seedlings were grown on 1/2 MS agar plates for 3 d and then on control or low Pi agar plates for 5 d. **F)** RT-qPCR analysis of *PHT1;1*, *PHT1;9*, and *PHO1;H1* in the rootstocks of grafted seedlings treated with Pi deficiency stress for 5 d. Expression levels of these genes were normalized first against the transcript level of *ACT2* and then against the values obtained from WT/WT grafted seedlings grown on control Pi agar plates. Data represent mean ± Sd of 6 **B)**, **C),** and **E)**; 5 **D);** and 4 **F)** biological replicates. Different letters above bars indicate statistically significant differences (*P* < 0.05 level; Tukey's multiple comparison test).

Our analyses indicated that Sec14-like proteins in chloroplasts are involved in the regulation of Pi uptake from the rhizosphere and in Pi deficiency responses in *Arabidopsis* and rice. To obtain further evidence supporting this conclusion, we generated *Arabidopsis* chimeras by grafting *atpitp7-2* scion on WT rootstock and vice versa and characterized their phenotypes. Like the *atpitp7-2* mutant, the chimeras constructed by grafting *atpitp7-2* scion on WT and *atpitp7-2* rootstock (*atpitp7-2*/WT and *atpitp7-2*/*atpitp7-2*, respectively) exhibited root growth defects (lower fresh root weight and primary root length) under both control and low Pi conditions compared with the WT/WT control chimera (Fig. 8, A to C). Furthermore, both Pi uptake activity and Pi content were lower in *atpitp7-2*/WT and *atpitp7-2*/*atpitp7-2* chimeras than in the WT/WT control chimera (Fig. 8, D and E), and Pi deficiency-inducible expression of *AtPHT1;1*, *AtPHT1;9*, and *AtPHO1;1* in roots was largely diminished in these chimeras (Fig. 8, F). By contrast, differences in growth, Pi uptake activity, Pi content, and Pi deficiency-inducible gene expression were not found between the WT/WT control chimera and WT/*atpitp7-2* graft chimera (Fig. 8). These results conclusively showed that reduced Pi uptake and Pi deficiency responses of *atpitp7-2* are the result of the loss of function of *AtPITP7* in shoots.

## Discussion

Functional characterization of *AtPITP7* and *OsPITP6* revealed that the inactivation of *AtPITP7* or *OsPITP6* caused growth defects under both Pi-sufficient and -deficient conditions, while overexpression of *AtPITP7* or *OsPITP6* enhanced Pi uptake activity and plant growth to a greater extent under Pi-deficient conditions than under Pi-sufficient conditions. Since homologs of AtPITP7 and OsPITP6 are likely to be present in other plant species (Supplemental Fig. S12), chloroplastic Sec14-like proteins may play a conserved and essential role, which is associated with the acquisition and effective utilization of Pi in response to Pi availability, in growth control in plants. Importantly, *OsPITP6*-overexpressing transgenic rice displayed agronomically superior traits due to the Pi-related function of OsPITP6. Therefore, it is worth emphasizing that chloroplastic Sec14-like proteins are useful for developing crops with improved growth under low Pi conditions.

### AtPITP7 as a potent factor influencing Pi uptake activity and Pi deficiency responses

Our GWAS of *Arabidopsis* accessions revealed an association of Pi uptake activity with *AtPITP7* but did not any association between Pi uptake activity and known Pi uptake- and Pi deficiency response-related genes for unknown reasons (Supplemental Fig. S1), suggesting that the chloroplastic Sec14-like protein AtPITP7 may be a potent factor influencing Pi deficiency responses. However, a very recent GWAS of Pi uptake activity in *Arabidopsis* accessions identified several genes associated with P-acquisition traits, including 4 Pi transporter genes, although it did not reveal the association between Pi uptake activity and *AtPITP7* (Chien et al. 2022). The reason for these conflicted results is currently unknown; however, Chien et al. discussed the possibility that their high-sensitive measurement method of Pi uptake activity might allow identifying Pi transporter genes as P-acquisition trait-associated genes. On the other hand, the association between *AtPITP7* and Pi uptake activity was identified in our GWAS, depending on only a few SNPs with −log10(*P*) > 4 (Supplemental Fig. S1). Therefore, different populations of *Arabidopsis* accessions used in GWASs might yield different results. Only some spontaneous mutations in *AtPITP7* alleles in a few *Arabidopsis* accessions might have a pronounced effect on Pi uptake activity.

### Chloroplastic Sec14-like proteins play an essential role in lipid remodeling in chloroplasts

Targeted metabolome analysis of membrane lipids provided evidence that OsPITP6 is involved in lipid remodeling in chloroplasts. In plants, Pi deficiency activates the eukaryotic pathway for galactolipid biosynthesis and promotes the conversion of PA to diacylglycerol (DAG) in extraplastidic membranes by phosphatidate phosphatase (PAP) to increase the flow of DAG, a precursor of MGDG and DGDG, to chloroplast membranes (Eastmond et al. 2010; Nakamura 2017). Hence, this eukaryotic pathway is indispensable for Pi recycling and lipid remodeling under Pi-deficient conditions (Nakamura 2013). Consistently, mutation of 2 *PAP* genes, *PAH1* and *PAH2*, in *Arabidopsis* caused severe defects in phospholipid turnover and led to excessive accumulation of PA and PC (Nakamura et al. 2009). The *pah1 pah2* double mutant *Arabidopsis* and *ospitp6* rice plants showed similar changes in lipid profiles compared with those in the WT plants (Fig. 6), suggesting that the growth defects in *ospitp6* plants may be due to the impaired Pi recycling and lipid remodeling.

However, unlike *ospitp6* plants, the *pah1 pah2 Arabidopsis* plants did not show alterations in PG and SQDG contents (Nakamura et al. 2009). PG is the most abundant phospholipid in chloroplasts (Hölzl and Dörmann 2019) and an anionic lipid necessary for the thylakoid membrane structure (Yu and Banning 2003), whereas SQDG is an anionic sulfolipid (also glycolipid) synthesized more actively under Pi-deficient conditions than under Pi-sufficient conditions. Thus, PG is substituted by SQDG under Pi-deficient conditions to maintain the abundance of anionic lipids in chloroplasts (Essigmann et al. 1998). SQDG is synthesized in the inner membrane of chloroplasts, and the last step of SQDG biosynthesis is catalyzed by SQDG synthase encoded by *SULFOQUINOVOSYLDIACYLGLYCEROL 2* (*SQD2*). In *Arabidopsis*, SQDG synthase produces SQDG and GlcADG, another anionic glycolipid, by transferring the sulfoquinovosyl moiety of UDP*-*sulfoquinovose or the glucuronic moiety of UDP-glucuronic acid to DAG, thus increasing both SQDG and GlcADG contents in *Arabidopsis* under Pi deficiency stress. Consistent with the importance of maintaining anionic lipid abundance under Pi deficiency stress, *Arabidopsis sqd2* mutant plants withered (Okazaki et al. 2013), and rice plants carrying a mutation in *OsSQD1*, which encodes UDP-sulfoquinovose synthase that catalyzes the conversion of UDP-glucose and sulfite (SO_3_^2−^) into UDP-sulfoquinovose, exhibited pale-green leaves and a severe Pi starvation phenotype (Sun et al. 2021) under Pi-deficient conditions. The precursor of DAG for SQDG production, PA, is provided not only through the eukaryotic pathway for glycolipid production but also through the prokaryotic pathway requiring glycerol 3-phosphate and acyl-CoA as precursors in chloroplasts. Proportional increases in SQDG and GlcADG contents during Pi deficiency were enhanced in *Ubi:OsPITP6* plants but reduced in *ospitp6* plants (Fig. 6), suggesting that chloroplastic Sec14-like proteins regulate the SQDG biosynthesis pathway rather than the eukaryotic pathway for galactolipid synthesis. Furthermore, since SQDG synthase is present in the chloroplast envelope (Cook et al. 2021), while OsPITP6 is localized in the stroma (Supplemental Fig. S15B), chloroplastic Sec14-like proteins might modulate Pi metabolism in chloroplasts because of its association with the regulation of the prokaryotic pathway. This hypothesis is supported by the significantly modified expression levels of *OsPT14* responsible for Pi import into chloroplasts (Shi et al. 2013) in shoots of *ospipt6* and *Ubi:OsPITP6* plants (Fig. 7, F; Supplemental Table S7). The possibility that chloroplastic Sec14-like proteins are additional regulators of Pi metabolism in chloroplasts is worth further investigation.

### Molecular basis of enhanced Pi uptake and improved Pi deficiency response in *Ubi:OsPITP6* plants

Transcriptome analysis revealed that the inactivation of *OsPITP6* affects a wide range of genes, including hormone-related genes (Fig. 7; Supplemental Fig. S24), suggesting that chloroplastic Sec14-like proteins are directly and indirectly involved in many physiological processes. As for Pi uptake-related genes, the inactivation of *OsPITP6* did not seem to reduce the expression levels of 2 *OsPT* genes (*OsPT1* and *OsPT2*), which are mainly responsible for Pi uptake from the rhizosphere (Liu et al. 2011), to low enough levels to explain the reduced activity of Pi uptake in *ospitp6* plants. This conflicts with the reduced Pi uptake in *ospitp6* plants. Since the activity of Pi transporters, especially PHT1 proteins, is regulated at the transcriptional, RNA stability, and posttranslational levels (Gu et al. 2016), the inactivation and overexpression of *OsPITP6* may influence Pi uptake activity, at least in part, through such posttranscriptional regulation. On the other hand, interestingly, the inactivation and overexpression of *OsPITP6* prominently altered the expression levels of *OsPT14*, which regulates Pi import into chloroplasts (Shi et al. 2013), as well as those of 3 *OsPHO1* genes involved in Pi translocation (Chiou 2020). Therefore, in *Ubi:OsPITP6* plants, Pi allocation under low Pi conditions might be modified, leading to improved Pi deficiency responses. Furthermore, since the phenotypes of grafted chimeras provided evidence that the *AtPITP7* allele in the aboveground plant parts is responsible for the phenotype of the *atpitp7* mutants (Fig. 8), overexpression of *OsPITP6* also may promote Pi uptake in roots via a long-distance regulatory mechanism. These mechanisms may contribute to enhanced Pi uptake and improved Pi deficiency responses in *Ubi:OsPITP6* plants. However, since OsPITP6 is the chloroplastic Sec14-like protein, it is difficult at this stage to precisely discuss the molecular basis of enhanced Pi uptake and improved Pi deficiency responses in *Ubi:OsPITP6* plants. Characterizing OsPITP6 as an additional regulator of Pi metabolism in chloroplasts may provide clues to understanding how overexpression of *OsPITP6* leads to enhanced Pi uptake and improved Pi deficiency responses in rice plants.

### Utility of chloroplastic Sec14-like proteins in developing crops with enhanced growth and yield under Pi deficiency stress

Improving crop production in Pi-depleted soils is 1 of the main objectives of plant breeding (López-Arredondo et al. 2014). Several genes capable of increased tolerance to Pi deficiency stress have been reported to date. For instance, P-starvation tolerance 1 (*PSTOL1*), which underlies the low Pi tolerance quantitative trait locus (QTL), Pup1, in rice and encodes the Pup1 protein kinase, has been shown to enhance grain yield in P-deficient soils (Gamuyao et al. 2012). Furthermore, overexpression of PHR1 enhanced Pi uptake in *Arabidopsis* (Nilsson et al. 2007). While our GWAS of *Arabidopsis* accessions revealed no association between Pi uptake activity and known Pi uptake- and Pi deficiency response-related genes for unknown reasons, it revealed an association of Pi uptake activity with *AtPITP7*. Therefore, genes encoding chloroplastic Sec14-like proteins may be a good target for biotechnology to enhance Pi deficiency responses. In fact, the overexpression of chloroplastic Sec14-like protein genes *AtPITP7* and *OsPITP6* similarly improved tolerance to Pi-deficient conditions in *Arabidopsis* and rice, respectively. Furthermore, each plant species is likely to possess its own chloroplastic Sec14-like protein gene. Thus, the use of chloroplastic Sec14-like protein genes could provide a promising genetic engineering approach for enhancing Pi acquisition and utilization efficiencies in various crops. This approach is quite different from the previously proposed genetic engineering approaches, including the manipulation of Pi transporter and key transcription factor genes and the secretion of organic acids and phosphatases (López-Arredondo et al. 2014). Furthermore, because *AtPITP7* was identified based on natural variation in its transcript level, the expression levels of chloroplastic Sec14-like protein genes may serve as a selection marker in conventional breeding programs. Hence, the identification of chloroplastic Sec14-like protein genes involved in Pi deficiency responses is expected to expand the opportunities for improving crop production in Pi-depleted soils substantially. It is worth mentioning that the GWAS did not reveal the significant association of Pi uptake activity with chloroplast function-related genes except for *AtPITP7* (Supplemental Fig. S1), emphasizing the unique role of chloroplastic Sec14-like proteins in the regulation of Pi uptake and Pi deficiency responses and their unique potential in biotechnological applications to improve crop production in Pi-depleted soils.

## Materials and methods

### Plant materials and growth conditions

*Arabidopsis* (*A. thaliana*) ecotype Columbia (Col-0) was used as the WT. Seeds of all *Arabidopsis* accessions, except Col-0 (Supplemental Table S1), and 2 T-DNA insertion mutants (*atpitp7-1*, SALK_047586C; *atpitp7-2*, SALK_116713C) were obtained from the *Arabidopsis* Biological Resource Center (ABRC), OH, United States of America. Seeds were sterilized, cold-stratified at 4 °C for 4 d, and sown on half-strength Murashige and Skoog (1/2 MS) agar plates (1/2 MS salts, 0.8% [*w*/*v*] agar, 0.5% [*w*/*v*] sucrose, and 3 mM MES-KOH [pH 5.8]). Seedlings were grown at 22 °C under continuous light in a growth chamber equipped with cool white fluorescent lamps (70 *μ*mol m^−2^ s^−1^).

Rice (*O. sativa* L.) cultivar Nipponbare was used as the WT. Sterilized rice seeds were germinated in deionized water under darkness at 37 °C for 3 d. Seedlings were grown first in 0.5× Yoshida nutrient solution (Yoshida et al. 1976), and then in Yoshida nutrient solution supplemented with 3 mM MES-KOH (pH 5.8) in a growth chamber maintained at 28 °C, long-day (14 h light/10 h dark) photoperiod, and 200 *μ*mol m^−2^ s^−1^ light intensity with cool white fluorescent lamps.

### Phenotypic analysis

To conduct phenotypic analysis, *Arabidopsis* seedlings were grown first on 1/2 MS agar plates for 5 d and then on control Pi or low Pi agar plates for 10 d. Low Pi agar plates contained low Pi medium (N-, P-, and Fe-free 1/2 MS salts; 2 mM KNO_3_; 2 mM NH_4_NO_3_; 1 *μ*M FeSO_4_; 0.5% sucrose; and 3 mM MES-KOH [pH 5.8]) and 0.8% agar. Pi concentration in low Pi agar plates was estimated to be ∼5 *μ*M Pi because of contamination from agar. Control Pi plates were prepared with low Pi medium supplemented with NaH_2_PO_4_ to a final concentration of 500 *μ*M. On the other hand, 3-d-old rice seedlings were grown in 0.5× Yoshida nutrient solution (Yoshida et al. 1976) for 7 d and then in control Pi nutrient solution (Yoshida nutrient solution supplemented with 3 mM MES-KOH [pH 5.8]) or low Pi nutrient solution (modified control Pi nutrient solution, in which NaH_2_PO_4_ concentration was reduced from 100 *μ*M to 2 *μ*M) for 25 d.

To analyze agricultural traits, 3-d-old rice seedlings were cultivated in regular soil (Honenagri Co. Ltd., Japan; N-sufficient soil) in a greenhouse until the reproductive stage. The greenhouse was set at 16 h light/8 h dark cycle, with supplemental lighting, and at 28 °C day/25 °C night temperature.

### Plasmid construction and plant transformation

*AtPITP7* cDNA obtained from the RNA of Col-0 seedlings was cloned between the cauliflower mosaic virus (CaMV) *35S* promoter and an MYC epitope tag-encoding sequence in the pGWP17 Gateway binary vector (Nakagawa et al. 2009). The resultant binary vector was verified by DNA sequencing and used for the transformation of *atpitp7-2* plants by the floral dip method (Zhang et al. 2006). The T2 progenies homologous for T-DNA insertion(s) introduced at a single locus were selected, and T3 and T4 homozygotes were used as *35S:AtPITP7/atpitp7-2* lines.

To generate *Ubi:OsPITP6* rice plants, the *NIGT1* cDNA located between the maize *Ubi* gene promoter and an MYC epitope tag-encoding sequence in pCB-HYG-ZmUbi-NIGT1-MYC (Sawaki et al. 2013) was replaced with the *OsPITP6* cDNA obtained using the RNA of Nipponbare seedlings. Transformation of the rice cultivar Nipponbare with the resultant binary vector, pCB-HYG-ZmUbi-OsPITP6-MYC, was carried out by INPLANTA INNOVATIONS Inc. (Yokohama, Japan). To edit the *OsPITP6* gene using the CRISPR/Cas9 system, 2 pairs of oligonucleotides (5′-GTTGCTCTCCTGGCGAGACCCCGC-3′ and 5′-AAACGCGGGGTCTCGCCAGGAGAG-3′; and 5′-GTTGGCGGGGTCTCGCCAGGAGAG-3′ and 5′-AAACCTCTCCTGGCGAGACCCCGC-3′) were first inserted into pU6gRNA. Then, DNA fragments obtained by the digestion of resultant plasmids with *Asc*I and *Pac*I were cloned into the CRISPR/Cas9 vector, pZH_OsU6gRNA_PubiMMCas9 (Mikami et al. 2015), to produce 2 single-guide RNAs (sgRNAs). Transformation of the rice cultivar Nipponbare with *Agrobacterium tumefaciens* (strain EHA105) harboring the constructed CRISPR/Cas9 vector, followed by the selection of transformed calli with antibiotics, was carried out as described in Toki et al. (2006). DNA sequencing of the target sites and the 3 possible off-target sites predicted by CRISPR-P v2.0 (http://crispr.hzau.edu.cn/CRISPR2/) was performed to confirm that the T0-generation plants of 3 independent lines carried biallelic mutations at the target locus but no undesired editing. T2 progenies of CRISPR/Cas9 lines homozygous for the introduced gene or the edited gene locus were obtained by self-pollination and used for further experiments. Primers used for plasmid construction are listed in Supplemental Table S9.

### Measurement of Pi uptake activity and Pi content

Pi uptake activity was measured in *Arabidopsis* seedlings grown on control Pi agar plates for 5 d and then on low Pi agar plates for 5 d, and in rice seedlings grown hydroponically in 0.5× Yoshida nutrient solution for 7 d and then in low Pi nutrient solution for 5 d. Images of ^33^P- or ^32^P-labeled Pi uptake were obtained using an FLA-5000 fluorescent image analyzer (Fujifilm, Japan), and the signal intensities of ^33^P and ^32^P were quantified using the ImageQuant TL software (GE Healthcare), as described previously (Sakuraba et al. 2018).

Pi contents were measured using 12-d-old *Arabidopsis* seedlings grown on 1/2 MS agar plates, grafted seedlings grown on control Pi agar plates, and rice seedlings grown in 0.5× Yoshida nutrient solution for 9 d. The frozen seedlings were ground to a fine powder, and ∼20 mg of the powder was suspended in 1 mL Milli-Q water. Pi concentration of the supernatant was measured using the ICS-3000 ion chromatography system (DIONEX Co.), according to the manufacturer's protocol.

### RT-qPCR analysis

First-strand cDNA was synthesized from total RNA extracted from *Arabidopsis* and rice seedlings using the Maxwell RSC Plant RNA Kit (Promega), SuperScript II reverse transcriptase (Invitrogen), and oligo(dT)_15_ primer. Then, RT-qPCR was conducted using the resultant cDNA, KAPA SYBR Fast qPCR Master Mix (KAPA biosystem), and gene-specific primers (Supplemental Table S9). Transcript levels of *Arabidopsis* and rice genes were normalized relative to those of *ACTIN2* (*ACT2*) and *UBIQUITIN 5* (*OsUBQ5*), respectively.

### Phylogenetic analysis

Nucleotide sequences of *AtPITP7* promoter regions were downloaded from the *Arabidopsis* 1001 Genomes database (http://signal.salk.edu). The amino acid sequences of *Arabidopsis* and rice Sec14-like proteins were obtained from The *Arabidopsis* Information Resource (TAIR) database (https://www.arabidopsis.org) and the Rice Annotation Project Database (RAP-DB) (https://rapdb.dna.affrc.go.jp/), respectively. A phylogenetic tree was constructed using the MEGA X software (https://www.megasoftware.net) by the neighbor-joining method with 1,000 bootstrap replicates.

### Protoplast transient assay

To conduct *Arabidopsis* protoplast transient assays, 6 reporter plasmids were constructed by cloning the Col-0-, Ws-0-, Ler-0-, Sorbo-, Rsch-4-, or Old-1-type *AtPITP7* promoter (equivalent to the region from −1,788 bp to +212 bp of the Col-0-type *AtPITP7* promoter, relative to the transcription start site) upstream of the luciferase gene in pJD301 (Luehrsen et al. 1992). Each reporter plasmid and an internal control plasmid carrying the β-glucuronidase gene (pUBQ10-GUS) were cotransfected into *Arabidopsis* protoplasts, according to the protocol of Yoo et al. (2007). LUC and GUS activities in each sample were determined using the Luciferase Assay System Kit (Promega) and 4-methylumbelliferyl β-D-glucuronide, respectively. LUC activity was normalized against GUS activity.

To perform rice protoplast transient assays, protoplasts were isolated from the leaf sheaths and stems of rice seedlings (Jung et al. 2015) and transformed with a plasmid, constructed by replacing the NLP7 cDNA in pHBT-NLP7-GFP-NOS (Liu et al. 2017) with the *OsPITP6* cDNA, according to the protocol of Yoo et al. (2007). The fluorescence signal of the OsPITP6-GFP fusion protein was observed using the BX51 fluorescence microscope (Olympus, Japan). Primers used for plasmid construction are listed in Supplemental Table S9.

### Quantification of chlorophyll pigments

*Arabidopsis* and rice leaf tissues were homogenized in 80% (*v*/*v*) ice-cold acetone with zirconia beads. Chlorophyll pigment extraction and quantification were carried out as described by Porra et al. (1989), respectively.

### Immunoblot analysis

Proteins in chloroplast subfractions were prepared as described by Keegstra and Yousif (1986). Total proteins in rice leaves were extracted using an extraction buffer (50 mM Tris-HCl [pH6.8], 10% glycerol [*v*/*v*], 2% SDS [*w*/*v*], 6% 2-mercaptoethanol [*v*/*v*], and 0.003% bromophenol blue [*w*/*v*]). The extracted proteins were separated on SDS-polyacrylamide gels, transferred onto an Immobilon-P transfer membrane (Merck Millipore), and detected using anti-MYC (Merck Millipore), -Lhcb1, -Lhca1, -α-tubulin, and -RbcL (Agrisera) antibodies, followed by an anti-mouse or anti-rabbit IgG HRP-linked antibody and Supersignal West Dura Extended Duration Substrate (Thermo Fisher Scientific).

### Detection of singlet oxygen

The accumulation of singlet oxygen in rice leaf blades was examined using the Singlet Oxygen Sensor Green (SOSG) reagent (ThermoFisher Scientific), as described previously (Piao et al. 2017). SOSG fluorescence was detected at 525 nm using the BX51 fluorescence microscope (Olympus, Japan) equipped with a digital camera (DP80, Olympus).

### Measurement of *Fv/Fm* ratio

To measure *Fv/Fm*, chlorophyll fluorescence images of rice leaves were captured and analyzed using the kinetics multispectral fluorescence imaging system (FluorCam 800 MF; Photon System Instruments, Czech Republic), according to the manufacturer's instructions.

### Measurement of C, N, and P contents

Three-day-old WT and *ospitp6-3* seedlings were grown in 0.5× Yoshida nutrient solution for 2 w and then in the control or low Pi nutrient solution for 2 w. Shoots and roots of the seedlings were separately collected and dried, and then, the C and N contents were analyzed by SI Science Co. Ltd. (Tokyo, Japan). For the measurement of P content, leaves and roots were collected separately and dried. Samples were finely ground in liquid N, and 100 mg of the resulting fine powder was digested in a mixture of 5 mL 98% (*w*/*w*) H_2_SO_4_ and 1 mL H_2_O_2_. After cooling to room temperature, the pH of the samples was adjusted to 5 using 5N NaOH. After removing cell debris by centrifugation, the P content was determined spectrophotometrically at 700 nm by the molybdenum blue method (Chiou et al. 2006).

### Quantification of phospholipids and glycolipids

Three-day-old WT, *ospitp6-3*, and *Ubi:OsPITP6* (L8) seedlings were grown in 0.5× Yoshida nutrient solution for 2 wk and then in the control or low Pi nutrient solution for 2 wk. Glycolipids in seedling leaves and intact chloroplasts were isolated as described previously (Mizusawa et al. 1999) and analyzed by liquid chromatography–tandem mass spectrometry (LC-MS/MS) after methylation with trimethylsilyldiazomethane, according to the protocol of Cai et al. (2016), with some modifications. Briefly, samples were frozen in liquid N, resuspended in tert-butyl methyl ether:methanol (1:1, *v*/*v*), stored overnight at −30 °C to inactivate endogenous lipases, and homogenized by a bead homogenizer. Then, tert-butyl methyl ether and 0.1 N HCl were added to each homogenate to obtain a final tert-butyl methyl ether:methanol:0.1 N HCl ratio of 10:3:3. After phase separation by centrifugation, the upper (ether) layer was recovered and dried in a vacuum concentrator. The residue was redissolved in the upper phase of tert-butyl methyl ether/methanol/0.01 N HCl (10:3:3) mixture, and the sample was then mixed with 1/10th volume of trimethylsilyldiazomethane (2.0 M in hexane). After 20 min incubation at room temperature, the reaction was stopped by adding 1/40th volume of acetic acid. Lipids were analyzed by the selected multiple reaction monitoring (MRM) modes using the LCMS-8030 system (Shimadzu, Japan), as described previously (Ukawa et al. 2022). Permethylated phospholipids were quantified using internal standards (14:0 PC, 14:0 PE, 16:0 PS, 18:0 PG, 18:0 PI, and 18:0 PA) purchased from Avanti Polar Lipids, United States of America. MGDG, DGDG, monomethylated SQDG, and monomethylated GlcADG were quantified by measuring relative peak intensity normalization with 18:0 PG peaks.

### Transcriptome analysis

Total RNA was extracted from the leaves and roots of 4-w-old soil-grown rice seedlings using the Maxwell RSC Plant RNA Kit (Promega). Preparation of cyanine-3-labeled cRNA using the Low Input Quick Amp Labeling Kit (One-Color; Agilent Technologies), hybridization with the Rice Oligo Microarray (V4; Agilent Technologies), and scanning of microarrays on the DNA Microarray Scanner (G2565BA; Agilent Technologies) were performed according to the manufacturer's instructions. Fluorescence signal intensities were detected using the Scan Control software (v7.0.03; Agilent Technologies). Data were extracted with the Feature Extraction software (v9.1; Agilent Technologies), and raw data were produced by calculating the average of 3 independent biological replicates. Welch's approximate Student's *t*-test was used for the pairwise comparison of different groups, and differences were considered significant at *P* < 0.05. Hierarchical average linkage clustering analysis of 16,110 genes differentially expressed between WT and *ospitp6*-3 plants (0.5 > fold change [FC] > 2) was performed using the *heatmap.2* package of R (ver. 4.0.3).

### Micrografting experiment

Scions and rootstocks were prepared from 5-d-old WT and *atpitp7*-*2* seedlings grown on 1/2 MS agar plates, placed on a 1/2 MS agar plate, and aligned under an optical microscope. After 4 d, the grafted seedlings forming connections between the scion and rootstock were transferred onto control Pi or low Pi agar plates and then grown for 5 d under continuous white light (70 *μ*mol m^−2^ s^−1^).

### Statistical analysis

All data shown in graphs are presented as the means ± standard deviation (Sd) from more than 4 biological replicates. Statistical significance was tested by 2-tailed Student's *t*-test (*P < 0.05, **P < 0.01) or Tukey's multiple comparison test (different lowercase letters indicate significant differences, *P* < 0.05).

### Accession numbers

Sequence data from this article can be found in the Arabidopsis Genome Initiative, the 1001 Genomes database, the TAIR database, or the Rice Annotation Project Database under these accession numbers: *AtACT2*, At3g18780; *AtPHO1;H1*, At1g68740; *AtPHT1;1*, At5g43350; *AtPHT1;9*, At1g76430; *AtPITP7*, At5g63060; *OsPHO1;1*, Os01g0110100; *OsPHO1;2*, Os02g0809800; *OsPHO1;3*, Os06g0493600; *OsPITP6*, Os02g0321500; *OsPT9*, Os06g0324800; *OsPT10*, Os06g0325200; OsPT14, Os02g0593500; and *OsUBQ5*, Os01g0328400. DNA microarray data were deposited in the Gene Expression Omnibus (GEO) repository of the National Center for Biotechnology Information (NCBI) database under the accession number GSE210819 on August 9, 2022.

## Supplementary Material

kiad212_Supplementary_Data

## Supplemental data

The following materials are available in the online version of the article.

**Supplemental Figure S1.** Manhattan plot representing the associations of single nucleotide polymorphisms (SNPs) with Pi uptake in *Arabidopsis*.

**Supplemental Figure S2.** Characterization of *atpitp7*-*1* and *atpitp7*-*2* mutants.

**Supplemental Figure S3.** Characterization of *35S:AtPITP7/atpitp7-2* plants.

**Supplemental Figure S4.** Similar Pi-related phenotypes of *atpitp7-1* and *atpitp7-2* seedlings.

**Supplemental Figure S5.** Phenotypes of WT, *pitp7-1*, and *pitp7-2* seedlings grown under different light conditions.

**Supplemental Figure S6.** Time-course analysis of *AtPITP7* expression under Pi deficiency.

**Supplemental Figure S7.** Phylogenetic analysis of *AtPITP7* promoter sequences in 102 *Arabidopsis* accessions.

**Supplemental Figure S8.** Alignment of AtPITP7 amino acid sequences of 5 *Arabidopsis* accessions.

**Supplemental Figure S9.** Positions of SNPs found at the *AtPITP7* locus among 18 *Arabidopsis* accessions.

**Supplemental Figure S10.** Comparison of AtPITP7 promoter sequences in clade IV accessions.

**Supplemental Figure S11.** Predicted subcellular localization of 32 Sec14-like proteins in *Arabidopsis*.

**Supplemental Figure S12.** Multiple sequence alignment of chloroplastic Sec14-like proteins in vascular plants.

**Supplemental Figure S13.** Phylogenetic relationships of *Arabidopsis* and rice Sec14-like proteins.

**Supplemental Figure S14.** Time-course analysis of *OsPITP6* expression during Pi deficiency treatment.

**Supplemental Figure S15.** Subcellular localization analysis of OsPITP6 protein.

**Supplemental Figure S16.** Nucleotide sequence of the target locus in *ospitp6-1*, *ospitp6-2*, and *ospitp6-3* plants.

**Supplemental Figure S17.** Characterization of *Ubi:OsPITP6* plants.

**Supplemental Figure S18.** Effects of the *ospitp6* knockout mutation on C, N, and P contents.

**Supplemental Figure S19.** Growth phenotype of *ospitp6-1*, *ospitp6-2*, and *ospitp6-3* seedlings.

**Supplemental Figure S20.** Differences in tiller number between WT and *Ubi:OsPITP6* plants.

**Supplemental Figure S21.** Expression analysis of *OsPITP6* in WT and *Ubi:OsPITP6* plants at different growth stages.

**Supplemental Figure S22.** Tiller numbers of WT, *ospitp6-3*, and *Ubi:OsPITP6* plants grown under low Pi conditions.

**Supplemental Figure S23.** Hierarchical average linkage clustering of 16,110 genes showing differential expression between WT and *ospitp6*-*3* seedlings.

**Supplemental Figure S24.** Alterations in the expression levels of phytohormone biosynthesis- and signaling-related genes in *ospitp6*-*3* plants.

**Supplemental Table S1.** *Arabidopsis* accessions used in the phylogenetic analysis.

**Supplemental Table S2.** Effects on C/N, C/P, and N/P ratios in rice shoots caused by Pi deficiency and the *ospitp6* mutation.

**Supplemental Table S3.** Top 50 genes upregulated in shoots of *ospitp6*-*3* seedlings.

**Supplemental Table S4.** Top 50 genes downregulated in shoots of *ospitp6*-*3* seedlings.

**Supplemental Table S5.** Top 50 genes upregulated in roots of *ospitp6*-*3* seedlings.

**Supplemental Table S6.** Top 50 genes downregulated in roots of *ospitp6*-*3* seedlings.

**Supplemental Table S7.** Actual values of signal intensities for expression levels of genes associated with Pi uptake, translocation, and signaling in shoots of WT and *ospitp6-3* seedlings.

**Supplemental Table S8.** Actual values of signal intensities for expression levels of genes associated with Pi uptake, translocation, and signaling in roots of WT and *ospitp6-3* seedlings.

**Supplemental Table S9.** Primers used in this study.
