# Supplementary material for: Chloroplastic Sec14-like proteins modulate growth and phosphate deficiency responses in Arabidopsis and rice
Source: Plant Physiol. 2023 Apr 6;192(4):3030–48. doi: 10.1093/plphys/kiad212 (PMC10400038; doi:10.1093/plphys/kiad212)
Supplement: kiad212_Supplementary_Data [file kiad212_supplementary_data.pdf]

Supplemental data

Chloroplastic Sec14-like proteins modulate growth and phosphate deficiency responses in Arabidopsis and rice

Mailun Yang[1], Yasuhito Sakruaba[1], Toshiki Ishikawa[2], Namie Ohtsuki[1], Maki Kawai-Yamada[2] and Shuichi Yanagisawa[1,*,‡]

1 Agro-Biotechnology Research Center, Graduate School of Agricultural and Life Sciences, The University of Tokyo, Tokyo 113-8657, Japan

2 Graduate School of Science and Engineering, Saitama University, Saitama 338-8570, Japan

[*]Author for correspondence: asyanagi@mail.ecc.u-tokyo.ac.jp

[‡]Senior author.

S.Y. conceived the project; M.Y., Y.S., and S.Y. designed the experiments; M.Y., Y.S., T.I., N.O., and M.K.-Y. performed the experiments; M.Y., Y.S., T.I., and S.Y. analyzed the data; and M.Y., Y.S., T.I, and S.Y. wrote the manuscript.

Running head: Chloroplastic Sec14-like protein

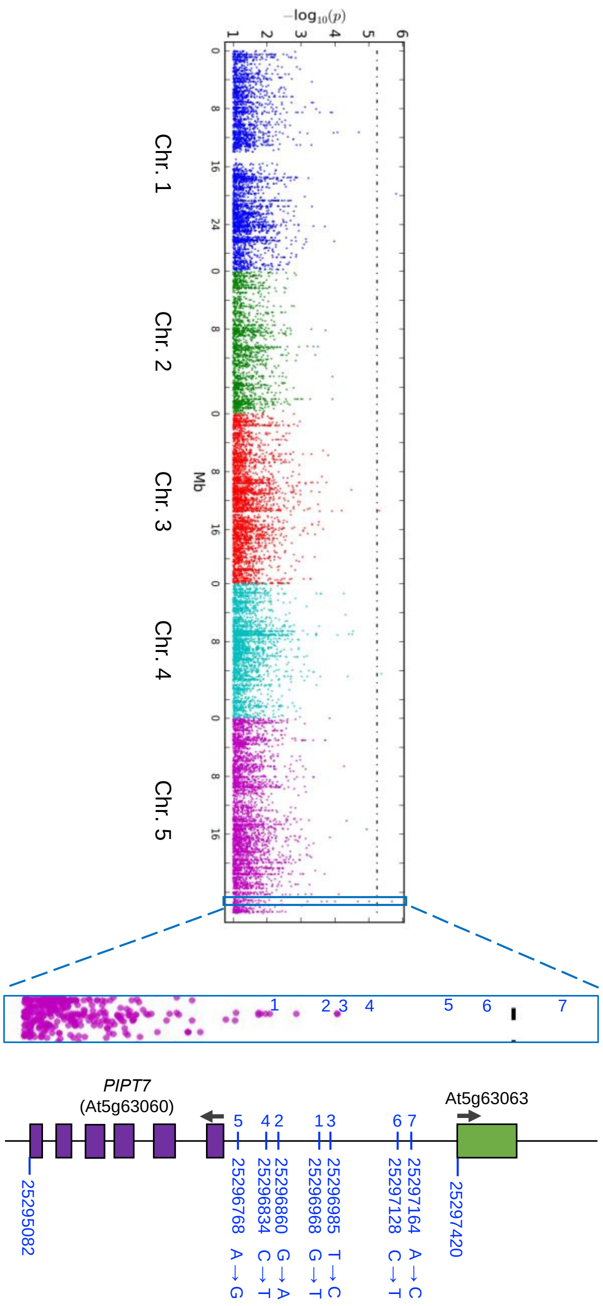

Supplemental Figure S1. Manhattan plot representing the associations of single nucleotide polymorphisms (SNPs) with Pi uptake in Arabidopsis. Each dot represents one SNP, and a dotted line indicates the position of *p*-value (0.05). SNPs with a small *p*-value were found within the *AtPITP7* locus.

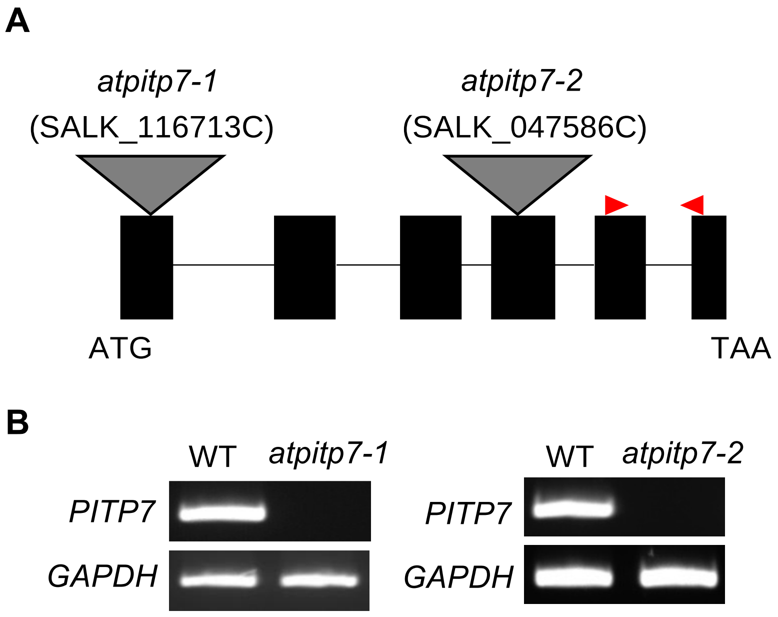

Supplemental Figure S2. Characterization of *atpitp7-1* and *atpitp7-2* mutants. A, Structure of *AtPITP7* (At5g63060). Black boxes and lines indicate coding regions and introns, respectively. T-DNA insertion positions in *atpitp7-1* (SALK_116713) and *atpitp7-2* (SALK_047586) and primer-binding sites are indicated by gray inverted triangles and red arrowheads, respectively. B, RT-PCR analysis of *AtPITP7* expression in the rosette leaves of WT, *atpitp7-1*, and *atpitp7-2* plants grown in soil for 15 d. *GAPDH* served as the internal control gene. PCR products were separated on an agarose gel and stained with ethidium bromide.

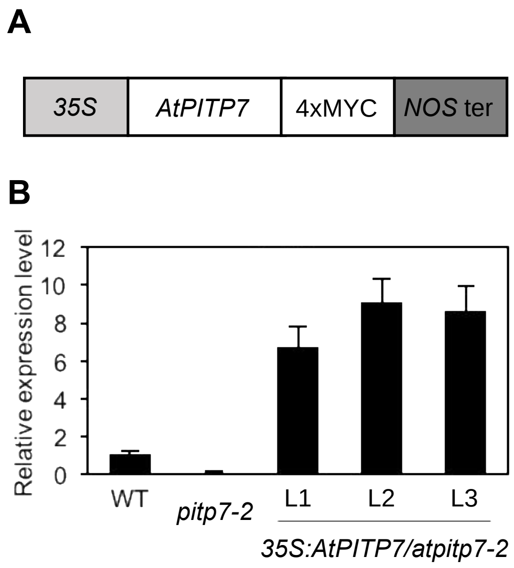

Supplemental Figure S3. Characterization of *35S:AtPITP7/atpitp7-2* plants. A, Schematic representation of the *35S:AtPITP7* construct used to generate transgenic Arabidopsis plants expressing the *AtPITP7-MYC* chimeric gene under the control of the *35S RNA* promoter in the *atpitp7-2* background. B, RT-qPCR analysis of *AtPITP7* expression in the rosette leaves of WT, *atpitp7-2*, and *35S:AtPITP7/atpitp7-2* (L1–L3) plants grown in soil for 15 d. Transcript levels of *AtPITP7* were normalized first against those of *ACT2* and then against the value obtained from WT rosette leaves. Data represent the mean ± standard deviation (SD) of four biological replicates.

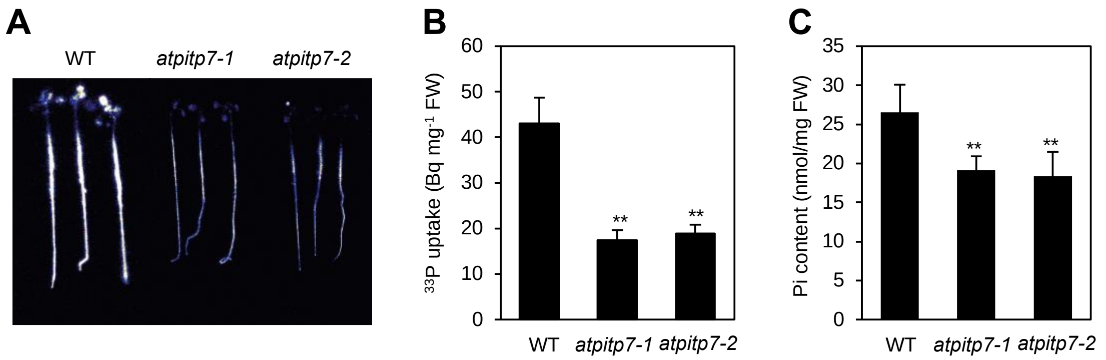

Supplemental Figure S4. Similar Pi-related phenotypes of *atpitp7-1* and *atpitp7-2* seedlings. A and B, Visualization (A) and quantification (B) of ^{33}P-labeled Pi uptake by 10d-old WT, *atpitp7-1*, and *atpitp7-2* seedlings grown on 1/2 MS agar plates for 5 d and then subjected to Pi deficiency treatment for 5 d. C, Quantification of Pi in 12d-old WT, *atpitp7-1*, and *atpitp7-2* seedlings grown on 1/2 MS agar plates. In B and C, data represent the mean ± SD of five biological replicates. Asterisks indicate significant differences compared to WT (***p* < 0.01; Student's *t*-test).

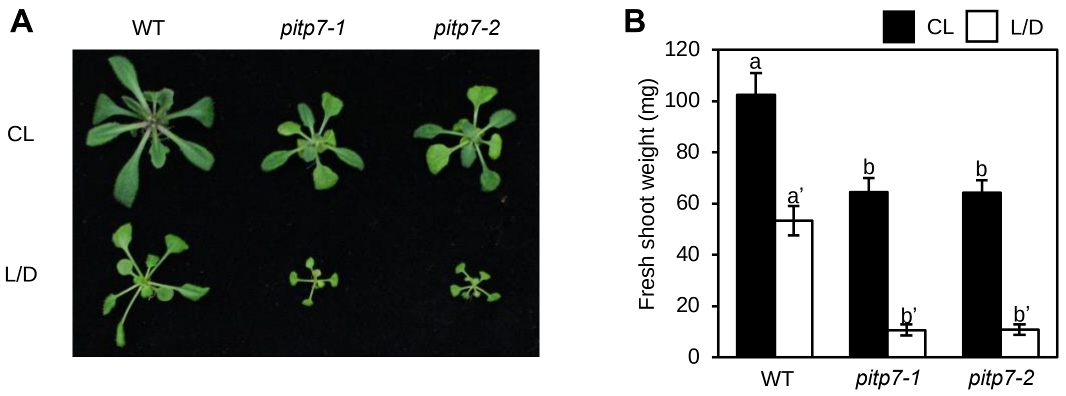

Supplemental Figure S5. Phenotypes of WT, *pitp7-1*, and *pitp7-2* seedlings grown under different light conditions. Photographs (A) and fresh shoot weight (B) of 3 w-old WT, *pitp7-1*, and *pitp7-2* seedlings grown on 1/2 MS medium supplemented with 1% sucrose under continuous light (CL) condition (70 μmol m^{-2} s^{-1}) or light/dark (L/D) condition (light 12 h, 80 μmol photons per m^{-2}·s^{-1} on leaf surfaces/dark 12 h) in a chamber set at 22 ℃. Different letters above bars indicate statistically significant differences ($p < 0.05$; Tukey's multiple comparison test).

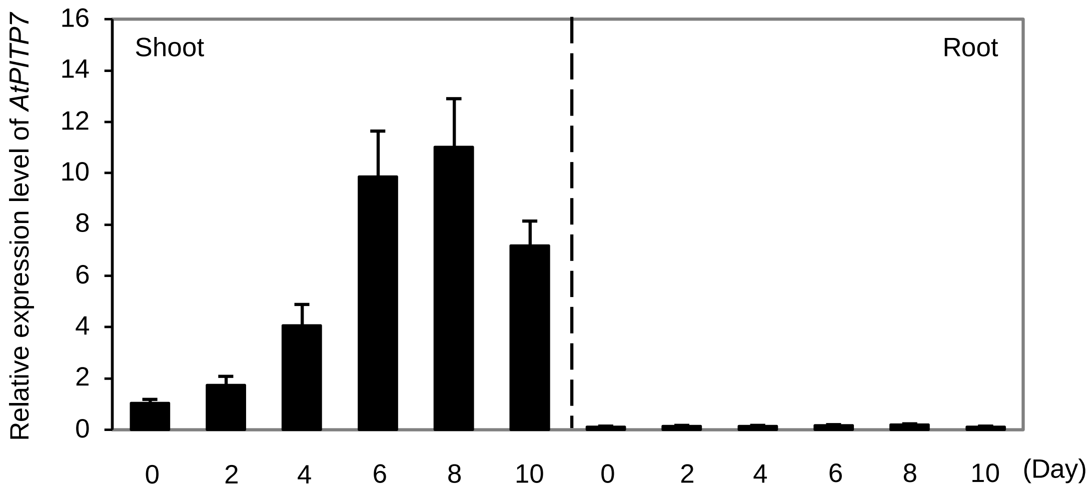

Supplemental Figure S6. Time-course analysis of *AtPITP7* expression under Pi deficiency. The expression of *AtPITP7* was analyzed by RT-qPCR in the shoots and roots of WT (Col-0) seedlings initially grown on 1/2 MS agar for 5 d (timepoint 0) and then grown under low Pi agar plates for the indicated days. *AtPITP7* transcript level was normalized first against the *ACT2* transcript level and then against the value obtained at the timepoint 0. Data represent the mean ± SD of four biological replicates.

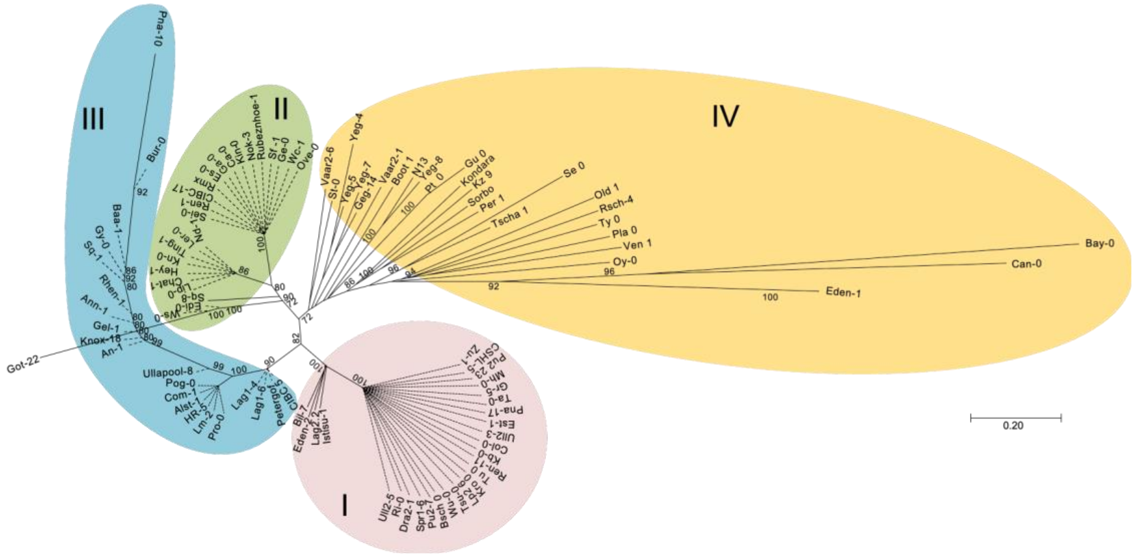

Supplemental Figure S7. Phylogenetic analysis of *AtPITP7* promoter sequences in 102 Arabidopsis accessions. A phylogenetic tree was constructed using the neighbor-joining (NJ) method. *AtPITP7* promoters are classified into four clades (I–IV) based on bootstrap values (indicated in percentages). Dotted lines indicate relationships that were enlarged without accurately reflecting distances and relationships. The scale bar corresponds to 0.20 estimated nucleotide substitutions per site. Numbers on the branches indicate the bootstrap percentage after 1000 replications in constructing the tree.

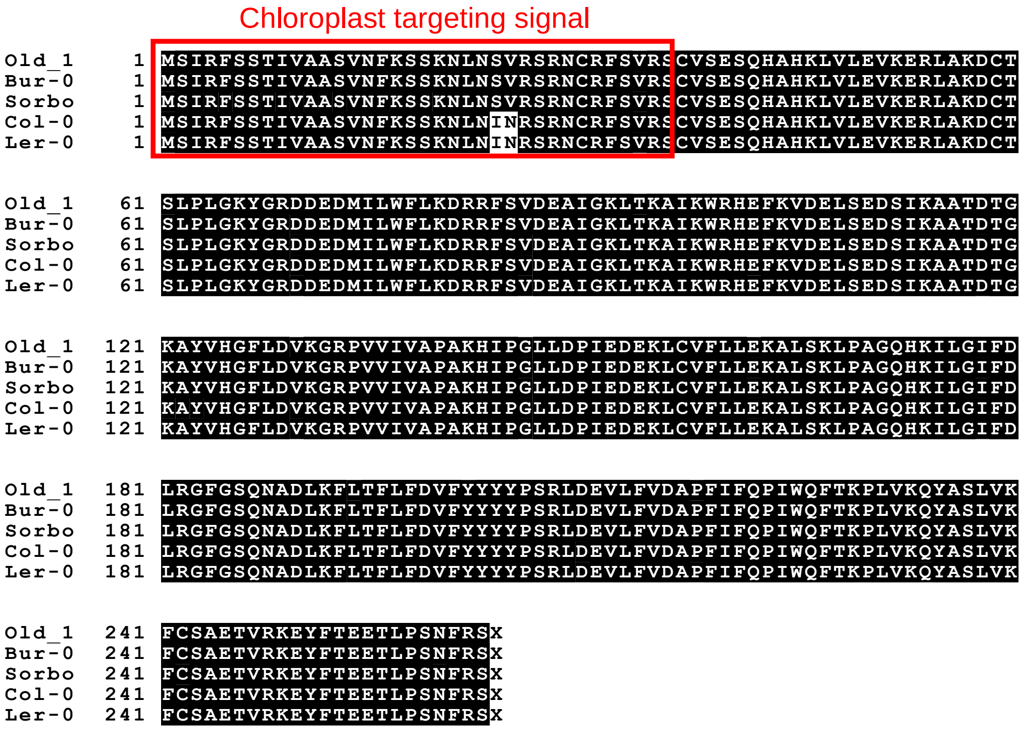

Supplemental Figure S8. Alignment of AtPITP7 amino acid sequences of five Arabidopsis accessions. Amino acid sequences of AtPITP7 of Old-1, Bur-0, Sorbo, Col-0, and Ler-0 were obtained from the 1001 Genomes database (https://1001genomes.org). Alignment was performed using CLUSTAL W (https://www.genome.jp/tools-bin/clustalw). Chloroplast targeting sequences predicted by targetP-2.0 (https://services.healthtech.dtu.dk/service.php?TargetP-2.0) are outlined with a red rectangle.

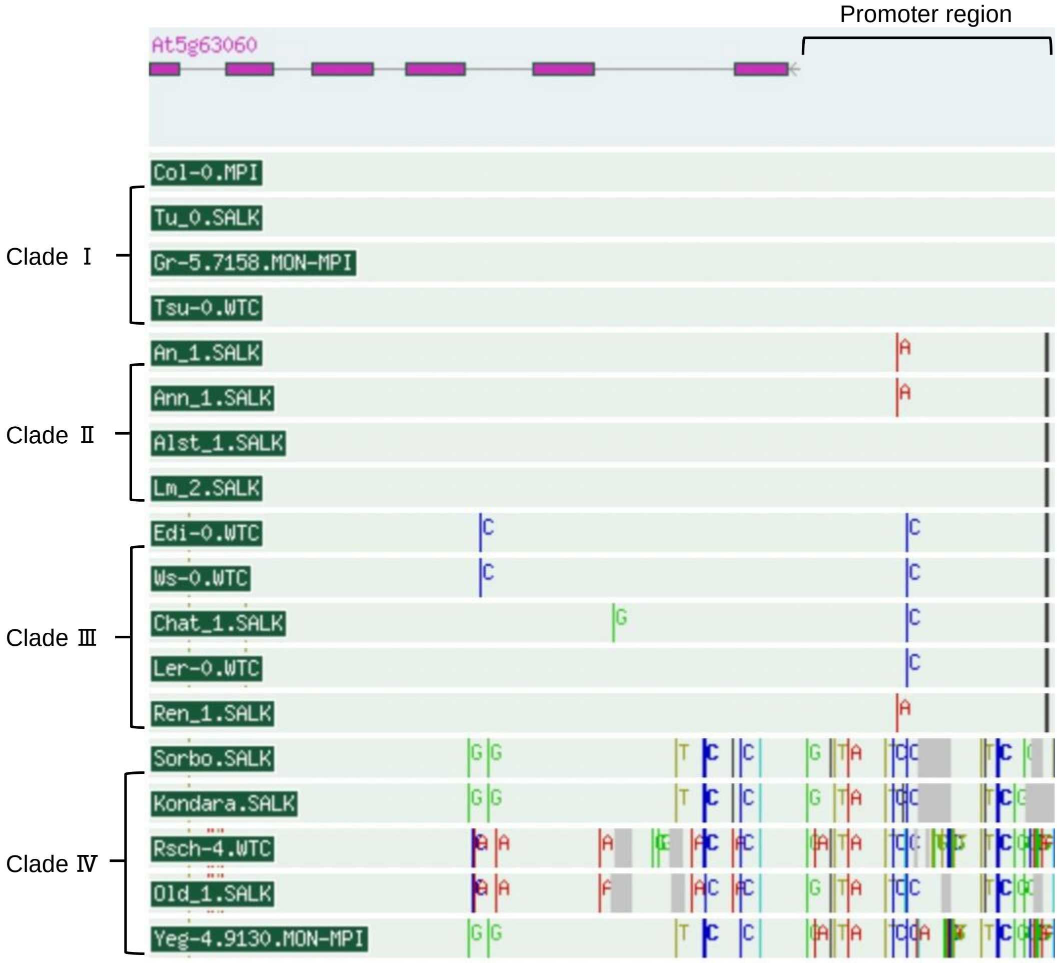

Supplemental Figure S9. Positions of SNPs found at the *AtPITP7* locus among 18 Arabidopsis accessions. Among the 18 accessions, four carried clade I *AtPITP7* promoter, four carried clade II promoter, five contained clade III promoter, and five harbored clade IV promoter. Nucleotide sequences of the *AtPITP7* locus of 18 Arabidopsis accessions were aligned in the 1001 Genomes database, and polymorphism information was obtained.

A

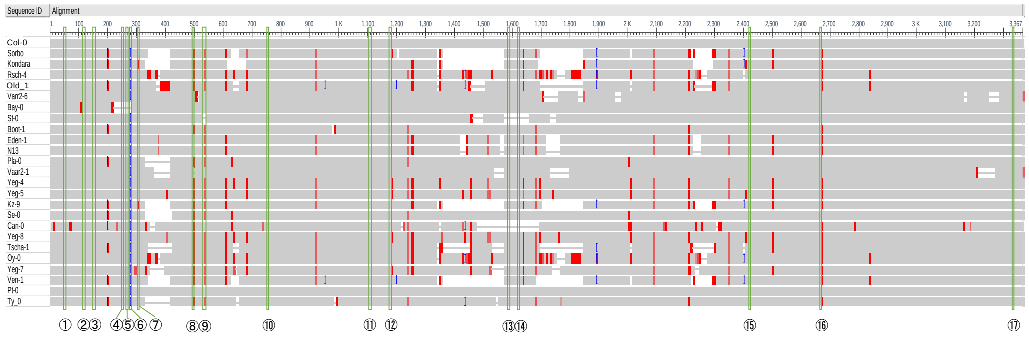

B

Number	The sequence in Col-0	The sequence in Clade Ⅳ
①	AAATTAGATTG	AAATTGGATTG
②	GAATTCAGAAT	GAATTTAGAAT
③	GAAGCGAAACA	GAAGCAAAACA
④	GTTCAGGGTCT	GTTCATGGTCT
⑤	CACGTTCGCAT	CACGTCCGCAT
⑥	GGTTTGGTAT	GGTTTGGTAAT
⑦	AAAATTTATTT	AAAATCTATTT
⑧	TAGGTCTTCTC	TAGGTTTTCTC
⑨	AAAACAAAATATCGT	AAAACCAAACATCGT
⑩	TTGTGGTGATG	TTGTGATGATG
⑪	CTCTGCTCCCC	CTCTGATCCCC
⑫	TACCTCGCACT	TACCTTGCACT
⑬	GCAAATGATCA	GCAAAGGATCA
⑭	TAATATGTGAA	TAATACGTGAA
⑮	AACAGAAAAAA	AACAGTAAAAA
⑯	ATCATGTTCCT	ATCATATTCCT
⑰	GGGAGGAAAAA	GGGAGAAAAAA

Supplemental Figure S10. Comparison of *AtPITP7* promoter sequences in clade IV accessions. A, Positions of polymorphisms present in the *AtPITP7* promoters of clade IV accessions. The *AtPITP7* promoter sequences were gathered from the 1001 Arabidopsis database and aligned with NCBI Multiple Sequence Alignment Viewer using the *AtPITP7* promoter of Col-0 as a reference. Red and blue bars indicate substitutions and insertions, respectively. White bars and boxes indicate deletions. Numbered green rectangles indicate mutations found in more than 70% of Clade Ⅳ accessions. B, Nucleotide sequences in the regions indicated by numbered green rectangles in A. Nucleotides that differ in Col-0- and Clade Ⅳ-type *AtPITP7* promoters are highlighted in yellow. The two putative *cis*-elements found in these sequences are shown in red and pink letters.

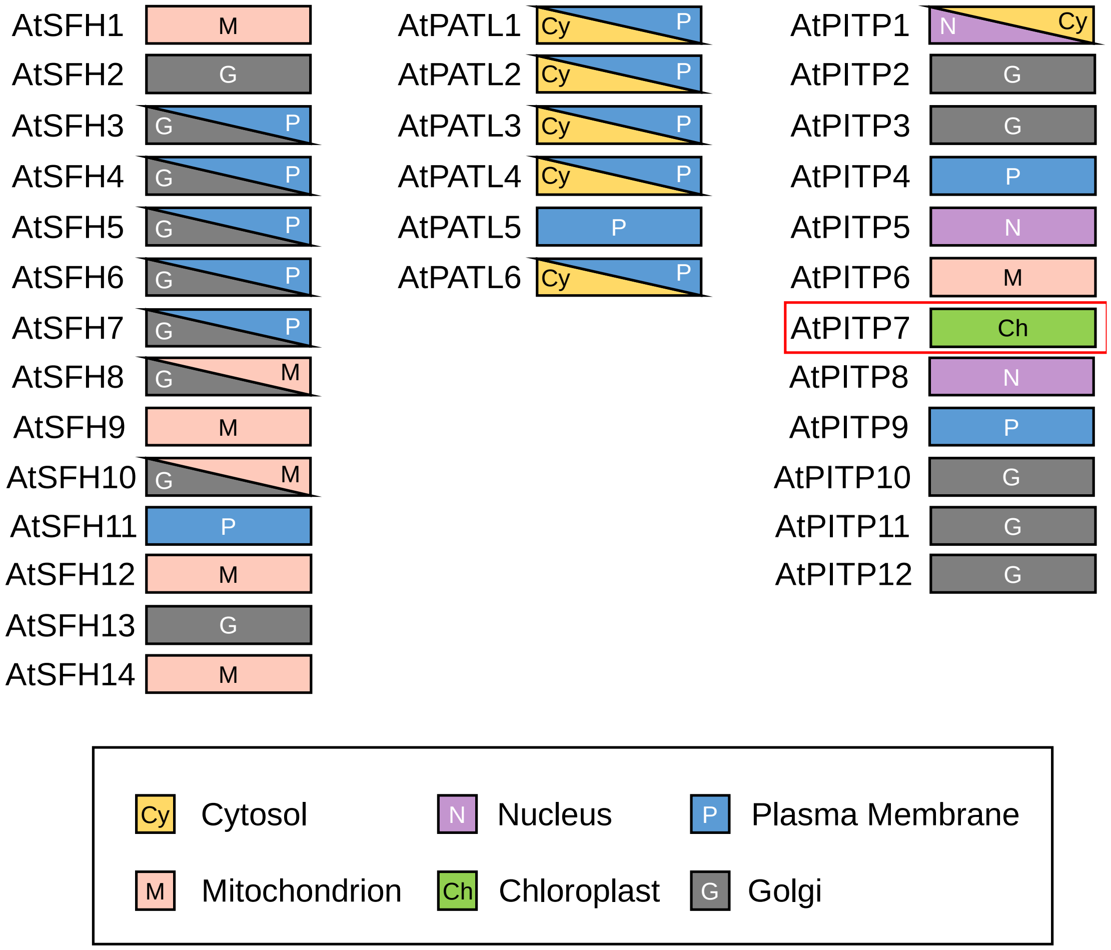

Supplemental Figure S11. Predicted subcellular localization of 32 Sec14-like proteins in Arabidopsis. Subcellular localization of each Sec14-like protein was predicted using the SUBA database (suba.live/siba-app/factsheet.html). The AtPITP7 protein indicated by a red rectangle is the only Sec14-like protein predicted to localize to chloroplasts.

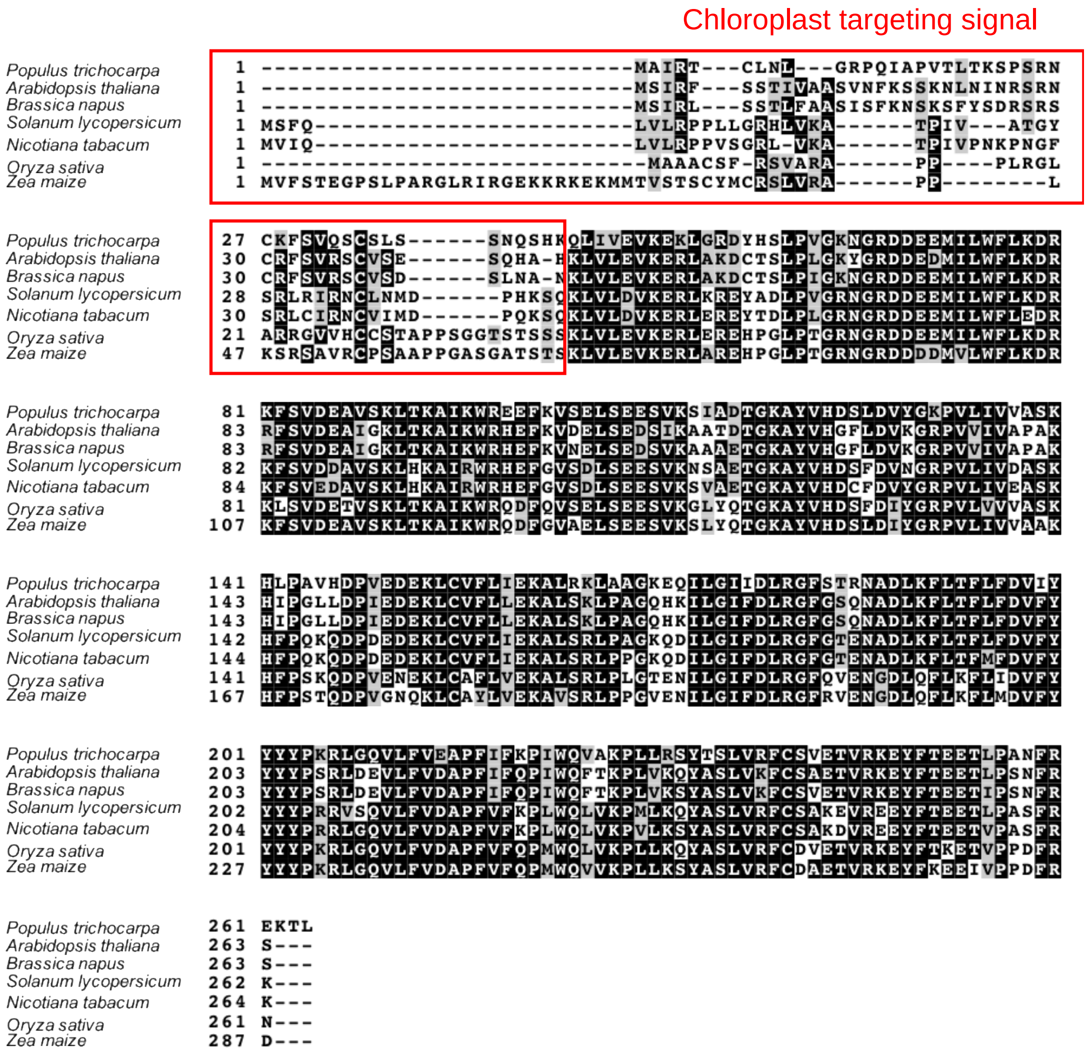

Supplemental Figure S12. Multiple sequence alignment of chloroplastic Sec14-like proteins in vascular plants. Chloroplastic Sec14-like proteins showing high homology to AtPITP7 and carrying a chloroplast targeting sequence were identified in *Populus trichocarpa*, *Brassica napus*, *Solanum lycopersicum*, *Nicotiana tabacum*, *Oryza sativa*, and *Zea mays* by a blast search against the NCBI database (https://www.ncbi.nlm.nih.gov/). The amino acid sequences obtained were aligned using CLUSTAL W (https://www.genome.jp/tools-bin/clustalw). Red rectangles indicate the putative chloroplast targeting sequences.

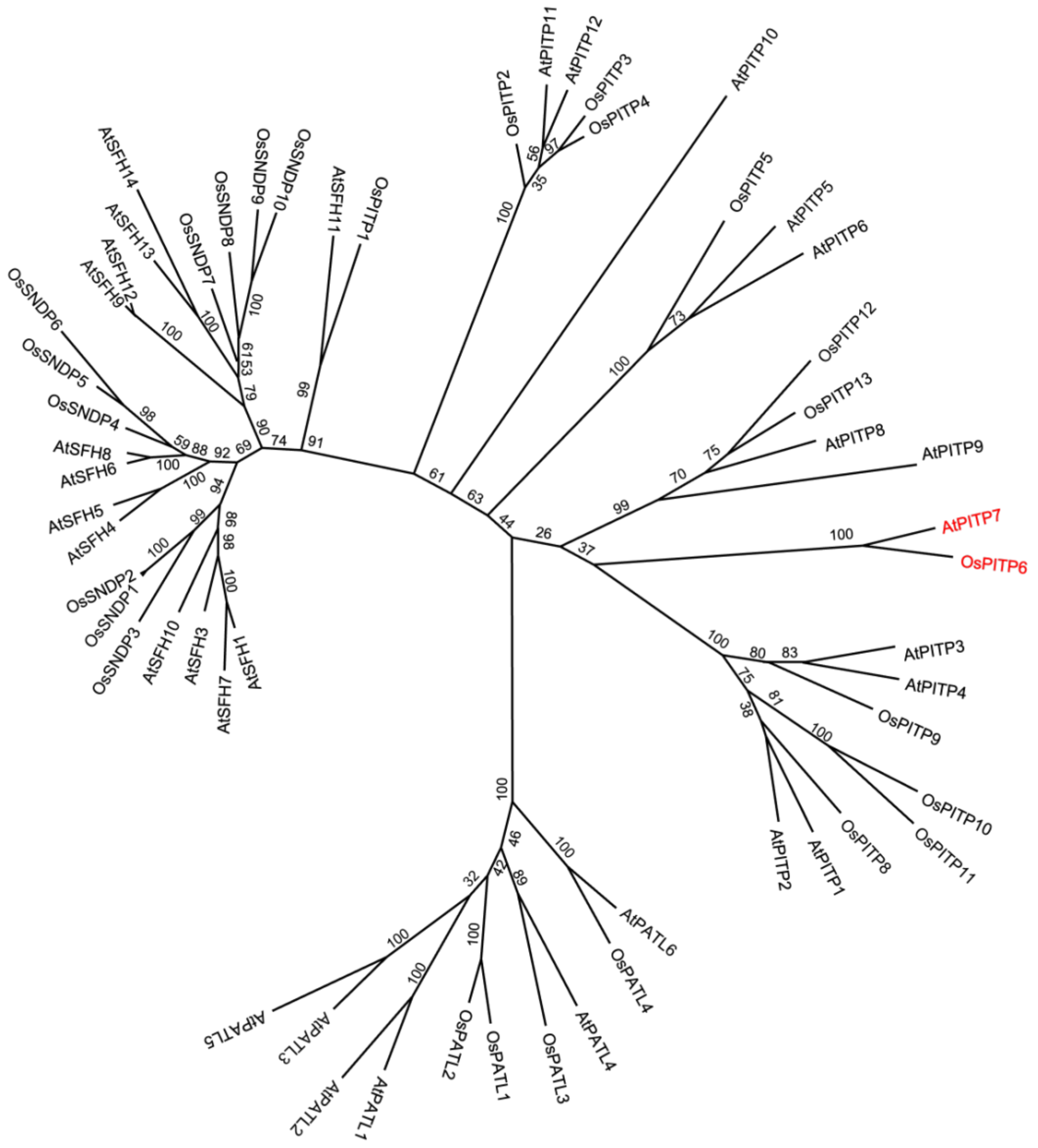

Supplemental Figure S13. Phylogenetic relationships of Arabidopsis and rice Sec14-like proteins. Amino acid sequences of Arabidopsis and rice Sec14-like proteins were downloaded from The Arabidopsis Information Resource (TAIR) database (https://www.arabidopsis.org/index.jsp) and RAP-DB (https://rapdb.dna.affrc.go.jp/), respectively. A phylogenetic tree was constructed by the neighbor-joining method using the MEGAX software with 1,000 bootstrap replicates. Bootstrap values are indicated in percentages. Arabidopsis and rice Sec14-like proteins with a chloroplast targeting sequence are indicated in red.

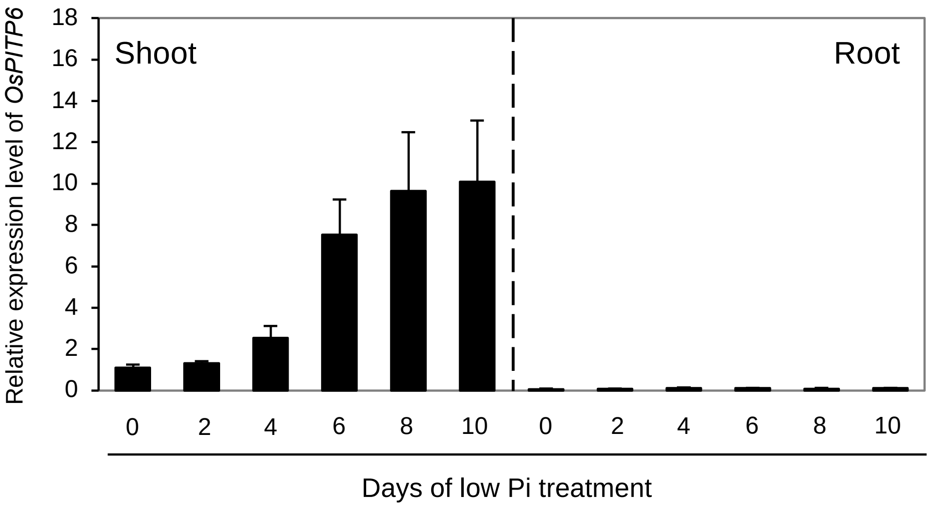

Supplemental Figure S14. Time-course analysis of *OsPITP6* expression during Pi deficiency treatment. RT-qPCR analysis of *OsPITP6* transcripts was performed using RNA extracted from shoots and roots of WT plants initially grown in the control Pi nutrient solution for 7 d and then in the low Pi nutrient solution for 2, 4, 6, 8, and 10 d. The expression of *OsPITP6* was normalized first against that of *OsUBQ5* and then against the value obtained at the 0 timepoint. Data represent the mean ± SD of four biological replicates.

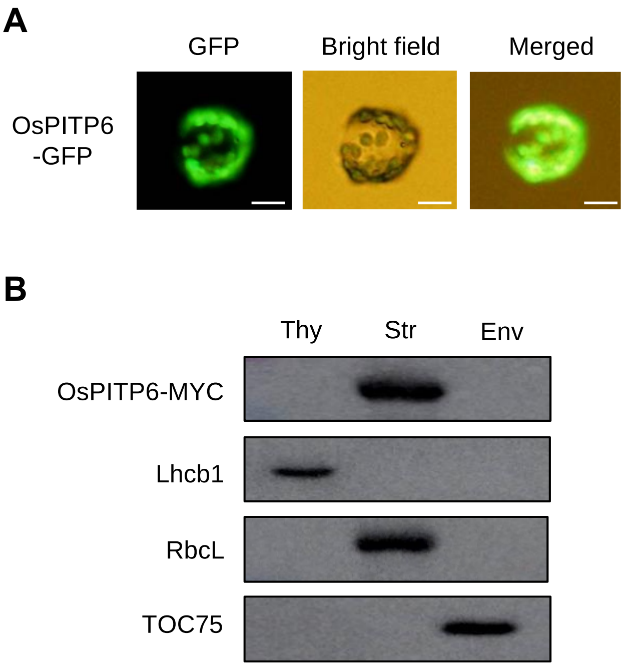

Supplemental Figure S15. Subcellular localization analysis of OsPITP6. A, Subcellular localization of the OsPITP6-GFP protein. The *OsPITP6-GFP* fusion was transiently expressed in protoplasts isolated from 2 w-old rice seedlings, and GFP fluorescence was observed by fluorescence microscopy. Scale bar = 10 nm. B, Immunoblot analysis of the OsPITP6-MYC protein in thylakoid (Thy), stroma (Str), and envelope (Env) fractions of chloroplasts. Intact chloroplasts were isolated from 45d-old soil-grown *Ubi:OsPITP6-MYC* plants. The purity of each fraction was verified with antibodies directed against thylakoid-, stroma-, and envelope-specific proteins (Lhcb1, RbcL, and TOC75, respectively). FW, fresh weight.

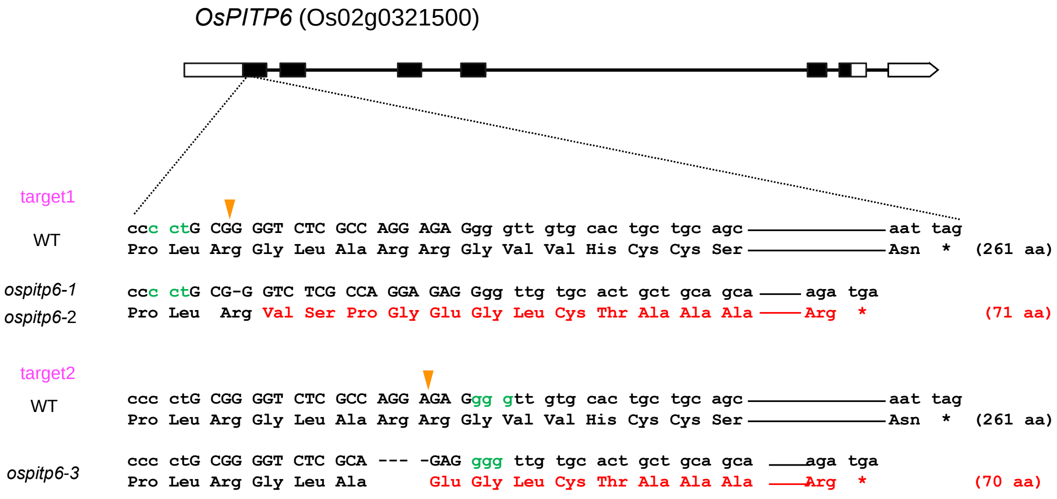

Supplemental Figure S16. Nucleotide sequence around the target locus of sgRNAs in *ospitp6-1*, *ospitp6-2*, and *ospitp6-3* plants. The position of the target region of sgRNAs is shown in the schematic representation of *OsPITP6*. Black squares indicate the coding region, while white squares and a white pentagon indicate untranslated regions. The target region of sgRNAs is indicated with capital letters. Two protospacer adjacent motif (PAM) sequences are indicated with green letters. Two putative cleavage sites are represented by orange triangles in the WT sequence. Hyphens (-) indicate deleted bases. The mismatched amino acids and stop codons generated by a frameshift are shown in red letters and stars, respectively. The numbers of amino acids in the predicted protein products of mutant alleles are also shown in red. The omission of nucleotide and amino acid sequences is indicated by solid lines. A 1 bp deletion induced by biallelic editing is predicted to generate a product of 71 amino acid residues in *ospitp6-1* and *ospitp6-2* plants, whereas a 4 bp deletion is predicted to generate a 70-amino acid product in *ospitp6-3*.

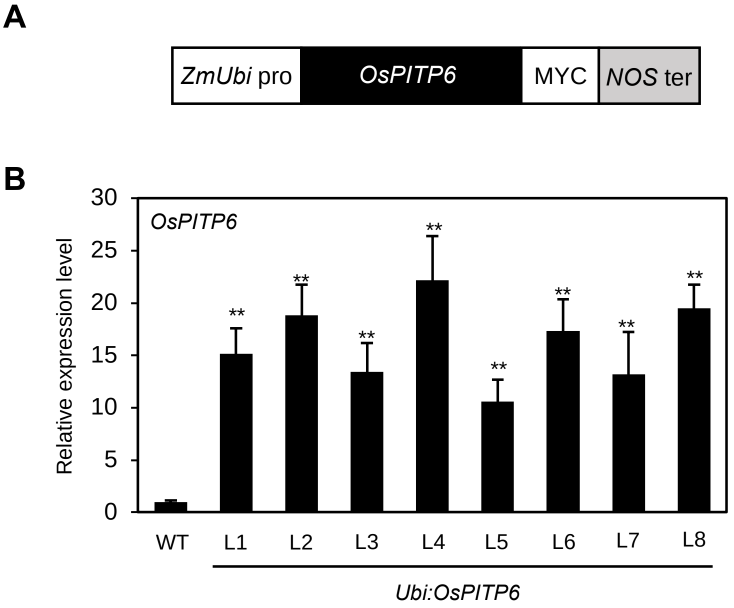

Supplemental Figure S17. Characterization of *Ubi:OsPITP6* plants. A, Schematic representation of the construct used for the generation of transgenic rice overexpressing the *OsPITP6-MYC* chimeric gene is expressed under the control of the maize ubiquitin (*Ubi*) gene promoter. B, RT-qPCR analysis of *OsPITP6* transcripts in the leaf blade of 3w-old WT plants and eight independent *Ubi:OsPITP6* transgenic rice lines (L1-L8). Levels of *OsPITP6* and *OsPITP6-MYC* transcripts were analyzed with the same PCR primers, and normalized first against the transcript level of *OsUBQ5* and then against the value obtained from the WT sample. Data represent the mean ± SD of four biological replicates, and asterisks indicate significant differences between WT and *ospitp6-3* samples (***p* < 0.01; Student's *t*-test).

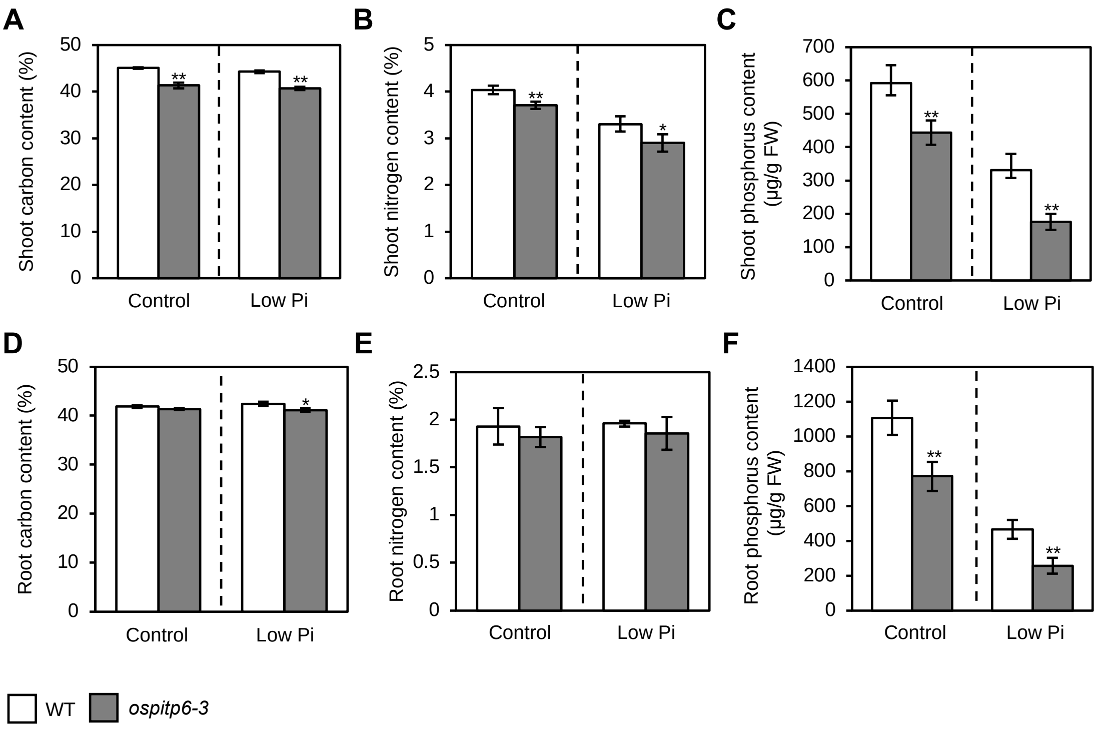

Supplemental Figure S18. Effects of the *ospitp6* knockout mutation on C, N, and P contents. C (A, D), N (B, E), and P (C, F) contents in shoots (A-C) and roots (D-F) were determined with WT and *ospitp6-3* seedlings that were grown in 0.5X Yoshida nutrient solution for 2 w and then in the control or low Pi nutrient solution for 2 w. C and N contents per dry weight and P content per fresh weight are shown. Data represent the mean ± SD of four biological replicates. Asterisks indicate significant differences between WT and *ospitp6-3* samples (**$p < 0.01$, *$p < 0.05$; Student's *t*-test).

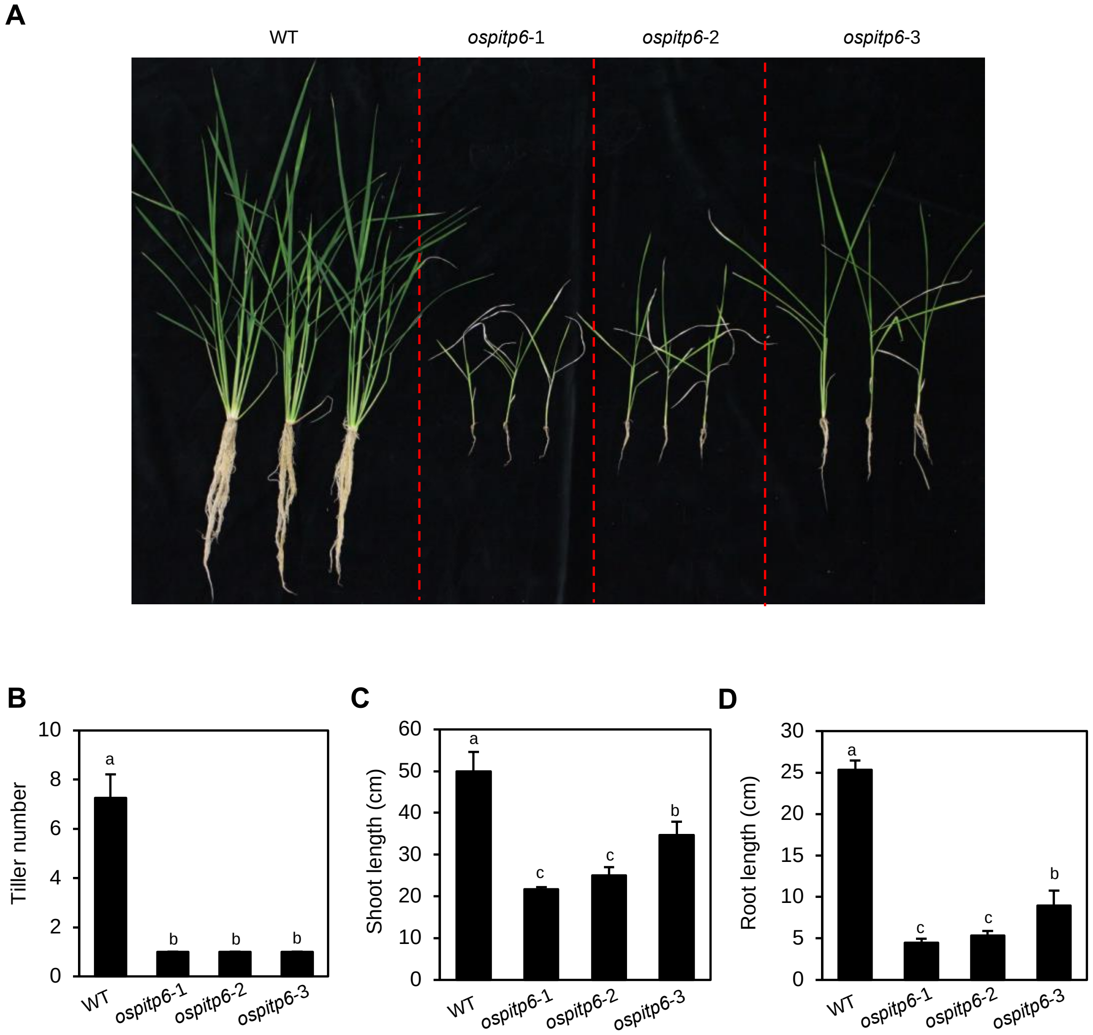

Supplemental Figure S19. Growth phenotype of *ospitp6-1, ospitp6-2*, and *ospitp6-3* seedlings. A-D, Photographs (A), tiller number (B), height (C), and root length (D) of 6w-old WT, *ospitp6-1, ospitp6-2*, and *ospitp6-3* seedlings grown in soil in the greenhouse. Data represent the mean ± SD of four biological replicates, and different letters above bars indicate statistically significant differences ($p < 0.05$; Tukey's multiple comparison test).

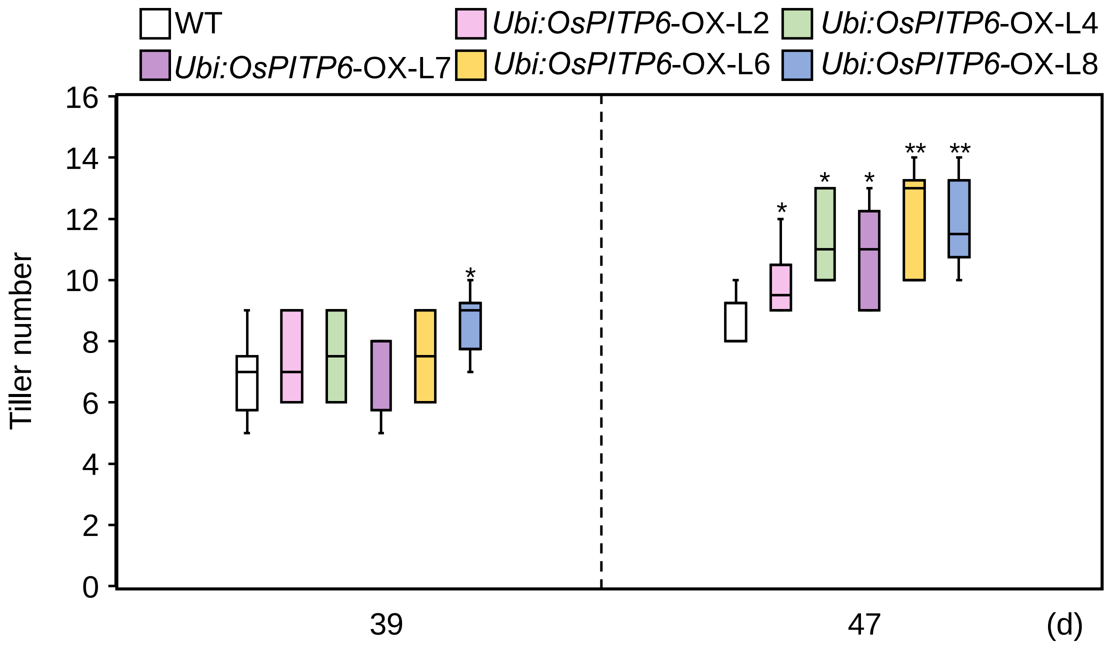

Supplemental Figure S20. Differences in tiller number between WT and *Ubi:OsPITP6* plants. Tiller numbers of 39d- and 47d-old soil-grown WT plants and five independent *Ubi:OsPITP6* transgenic lines are shown. Asterisks indicate significant differences between WT and *Ubi:OsPITP6* lines (**$p < 0.01$, *$p < 0.05$; Student's *t*-test). Horizontal lines in the box denote median values. Boxes extend from the 25th percentile to the 75th percentile of each line's distribution of values. Asterisks indicate significant differences between WT and *Ubi:OsPITP6* samples (*$p < 0.05$, **$p < 0.01$; Student's *t*-test).

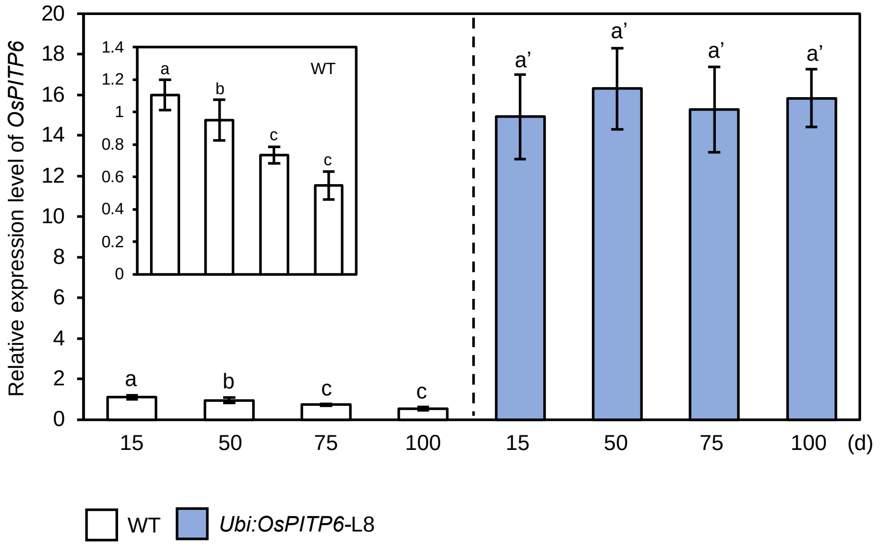

Supplemental Figure S21. Expression analysis of *OsPITP6* in WT and *Ubi:OsPITP6* plants at different growth stages. Transcript levels of *OsPITP6* were analyzed by RT-qPCR in shoots of 15d-, 50d-, 75d-, and 100d-old WT and *Ubi:OsPITP6* plants grown in soil. *OsPITP6* transcript levels were normalized first against *UBQ5* transcript levels and then against the value obtained with 15d-old WT plants. Data represent the mean ± SD of four biological replicates, and different letters above bars indicate statistically significant differences ($p < 0.05$; Tukey's multiple comparison test).

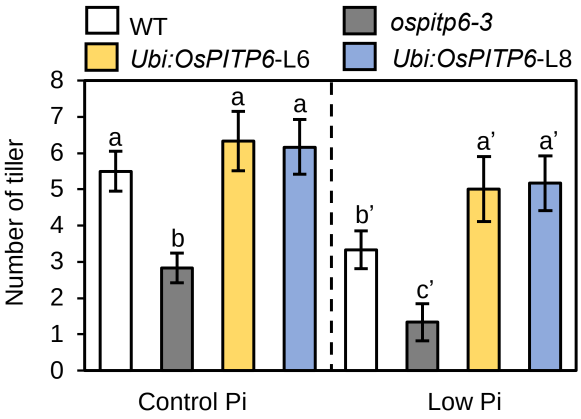

Supplemental Figure S22. Tiller numbers of WT, *ospitp6-3*, and *Ubi:OsPITP6* plants grown under low Pi conditions. Twenty-one-day-old seedlings were grown in the control and low Pi nutrient solution for 15 d. Different letters above bars indicate statistically significant differences ($p < 0.05$; Tukey's multiple comparison test).

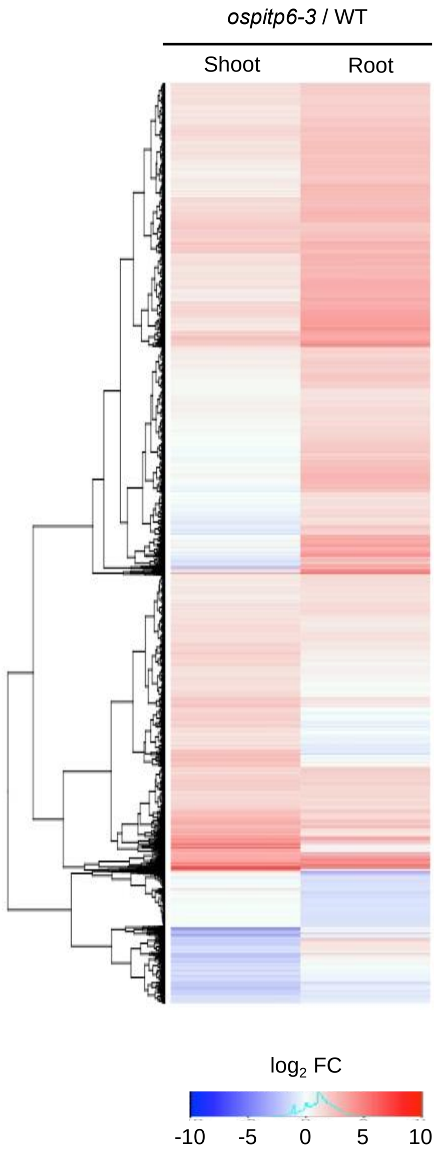

Supplemental Figure S23. Hierarchical average linkage clustering of 16,110 genes showing differential expression between WT and *ospitp6-3* seedlings. Left and right rows represent the log-transformed values of fold change (log_2FC) in the expression level of each gene (transcript level in *ospitp6-3* relative to that in the WT) in shoots and roots, respectively. Green and red columns represent downregulated genes (FC < 0.5) and upregulated genes (FC > 2), respectively.

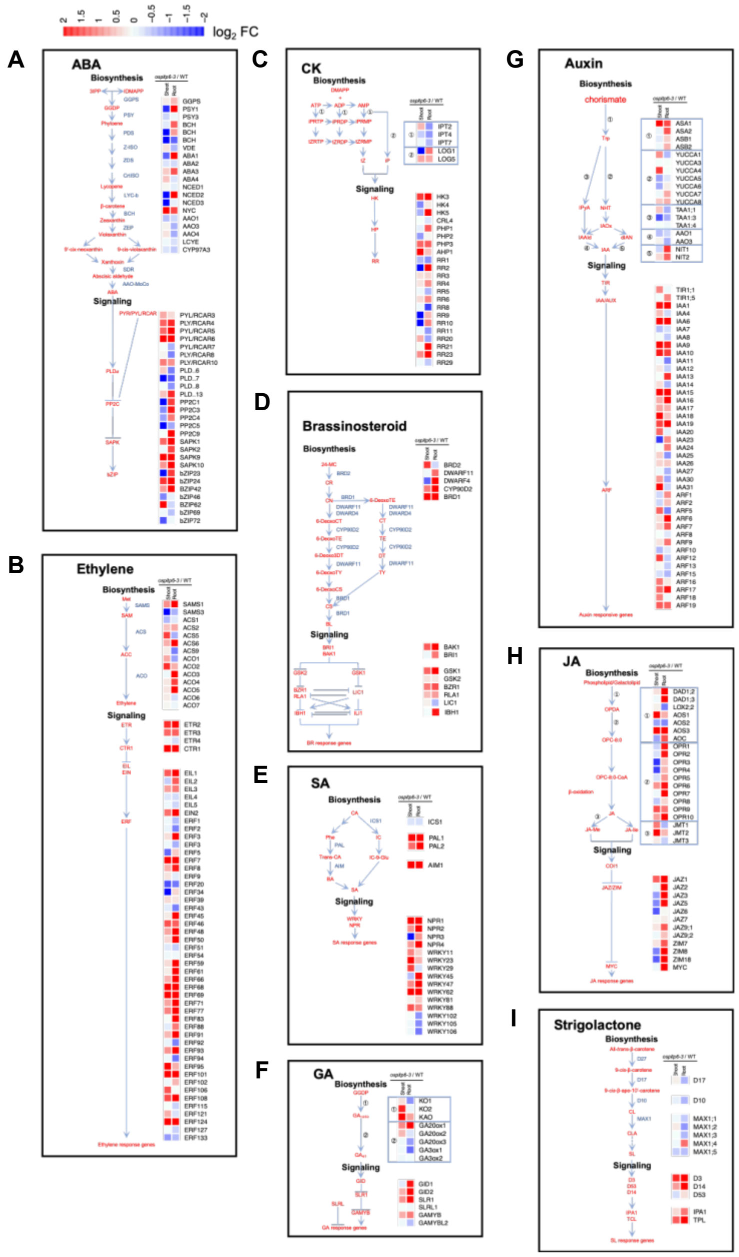

Supplemental Figure S24. Alterations in the expression levels of phytohormone biosynthesis- and signaling-related genes in *ospitp6-3* plants. A-I, *ospitp6-3* allele-dependent changes (log_2FC) in the expression levels of genes associated with the signaling and biosynthesis of abscisic acid (ABA) (A), ethylene (B), cytokinin (CK) (C), brassinosteroid (BR) (D), salicylic acid (SA) (E), gibberellin (GA) (F), auxin (G), jasmonic acid (JA) (H), and strigolactone (I). Information on phytohormone-associated genes was obtained from RAP-DB (https://rapdb.dna.affrc.go.jp/).

Supplemental Table S1 Arabidopsis accessions used in the phylogenetic analysis.

Accession	ABRC stock ID	Country	Latitude	Longitude
Sorbo	CS28742	TJK	38.35	68.48
Kondara	CS28418	TJK	38.48	68.49
Kb-0	CS28380	GER	50.1797	8.50861
Ge-0	CS1187	SUI	46.5	6.08
Rsch-4	CS1494	RUS	56.3	34
Rmx-A180	CS28690	USA	42.036	-86.511
Est-1	CS22683	RUS	58.3	25.3
Old-1	CS6820	GER	53.1667	8.2
Bur-0	CS22679	IRL	53.08	-9.07555556
Var-6	CS22581	SWE	55.58	14.334
Bay-0	CS22676	GER	49	11
Lip-0	CS1337	POL	50	19.3
Pna-17	CS28647	USA	42.0945	-86.3253
NFA-10	CS28533	UK	51.4083	-0.6383
Hs-0	CS1237	GER	52.24	9.44
ST-0	CS1535	SWE	59	18
Dra-2-1	CS6687	SWE	55.76	14.12
Boot-1	CS22551	UK	54.4	-3.2667
Ca-0	CS6658	GER	50.2981	8.26607
Eden-1	CS28218	SWE	62.877	18.177
N13	CS22491	RUS	61.36	34.15
Rhen-1	CS22536	NED	51.9667	5.56667
Got-22	CS22609	GER	51.5338	9.9355
Bu-0	CS1007	GER	50.5	9.5
Ull2-3	CS28791	SWE	56.0648	13.9707
Kin-0	CS28388	USA	44.46	-85.37
Kn-0	CS6762	LTU	54.8969	23.8924
Pla-0	CS6834	ESP	41.5	2.25
Wc-1	CS28813	GER	52.6	10.0667
Var2-1	CS22580	SWE	55.58	14.334

Yeg-4	CS22768	ARM	39.8692	45.3622
Ren-11	CS22611	FRA	48.5	-1.41
Edi-0	CS28221	UK	55.9494	-3.16028
Yeg-5	CS22769	ARM	39.8692	45.3622
Kz-9	CS22607	KAZ	49.5	73.1
Pu2-7	CS28654	CZE	49.42	16.36
An-1	CS28016	BEL	51.2167	4.4
Col-0	CS22681	USA	38.3	-92.3
Pog-0	CS6842	CAN	49.2655	-123.206
Se-0	CS28727	ESP	38.3333	-3.53333
Can-0	CS1065	ESP	29.2144	-13.4811
Knox-18	CS22567	USA	41.2816	-86.621
Yeg-8	CS22772	ARM	39.8692	45.3622
Com-1	CS22522	FRA	49.416	2.823
Sq-8	CS28748	UK	51.4083	-0.6383
Ta-0	CS1549	CZE	49.5	14.5
Tu-0	CS1567	ITA	45	7.5
Tscha-1	CS22518	AUT	47.0748	9.9042
Cibc-5	CS22602	UK	51.4083	-0.6383
Gy-0	CS28334	FRA	49	2
Oy-0	CS28592	NOR	60.385543	6.193019
Gel-1	CS6718	NED	51.0167	5.86667
Pro-0	CS28652	ESP	43.28	-6.01
Ws-0	CS915	RUS	52.3	30
Sf-1	CS6855	ESP	41.7833	3.03333
Yeg-7	CS22771	ARM	39.8692	45.3622
Ann-1	CS22520	FRA	45.9	6.13028
Rubezhone-1	CS927	UKR	49	38.28
Gr-5	CS28326	AUT	47	15.5
Pna-10	CS28646	USA	42.0945	-86.3253
Petergof	CS926	RUS	59	29
Kro-0	CS6766	GER	50.0742	8.96617

Chat-1	CS22521	FRA	48.0717	1.33867
Mh-0	CS6792	POL	50.95	20.5
Ven-1	CS22538	NED	52.0333	5.55
Geg-14	CS22763	ARM	40.1408	44.8203
Ove-0	CS28590	GER	53.3422	8.42255
Ting-1	CS22549	SWE	56.5	14.9
Spr1-6	CS28745	SWE	56.3163	16.0353
Lp2-6	CS28480	CZE	49.38	16.81
Eden-2	CS28219	SWE	62.877	18.177
Bil-7	CS28077	SWE	63.324	18.484
Nd-1	CS28531	GER	50	10
Ler-0	CS22686	GER	47.984	10.8719
Sq-1	CS28746	UK	51.4083	-0.6383
Pu2-23	CS22454	CZE	49.42	16.36
Hey-1	CS22534	NED	51.25	5.9
Es-0	CS6699	FIN	60.1997	24.5682
Bil-5	CS28076	SWE	63.324	18.484
Alst-1	CS22550	UK	54.8	-2.4333
Lag1-6	CS22742	GEO	41.8296	46.2831
Baa-1	CS22529	NED	51.3333	6.1
Ullapool-8	CS28972	UK	57.9	-5.1525
Pt-0	CS28653	GER	53.476	10.6065
Pu2-8	CS22449	CZE	49.42	16.36
Ren-1	CS22610	FRA	48.5	-1.41
Gu-0	CS22617	GER	50.3	8
HR-5	CS22596	UK	51.4083	-0.6383
Istisu-1	CS28362	AZE	38.9786	48.5594
Nok-3	CS28571	NED	52.24	4.45
Ull-2-5	CS28792	SWE	56.0648	13.9707
Ty-0	CS6878	UK	56.4278	-5.23439
Zu-1	CS6903	SUI	47.3667	8.55
Lag2-2	CS22747	GEO	41.8296	46.2831

Ri-0	CS28686	CAN	49.1632	-123.137
Tsu-0	CS6874	JPN	34.43	136.31
Ga-0	CS28273	GER	50.3	8
Wu-0	CS28838	GER	49.7878	9.9361
Lag1-4	CS22740	GEO	41.8296	46.2831
Per-1	CS1445	RUS	58	56.3167
CIBC-17	CS22603	UK	51.4083	-0.6383
Sei-0	CS6853	ITA	46.5438	11.5614
Bsch-0	CS6630	GER	50.0167	8.6667
CSHL-5	CS22423	USA	40.8585	-73.4675
Lm-2	CS1345	FRA	48	0.5

Supplemental Table S2. Effects on C/N, C/P, and N/P ratios in rice shoots caused by Pi deficiency and the *ospitp6* mutation.

Ratio	Shoot				Root			
	Control		Low Pi		Control		Low Pi	
	WT	*ospitp6-3*	WT	*ospitp6-3*	WT	*ospitp6-3*	WT	*ospitp6-3*
C/N	1.00	1.00	1.20	1.26	1.00	1.05	1.00	1.02
C/P	1.00	1.23	1.76	3.05	1.00	1.42	2.40	4.25
N/P	1.00	1.23	1.46	2.47	1.00	1.36	2.41	4.16

[a]C and N contents are divided by N or P contents and then shown relative to the values obtained with shoots or roots of WT seedlings grown under the control conditions.

Supplemental Table S3 Top 50 genes upregulated in shoots of *ospitp6-3* seedlings.

Locus ID	Description	Fold	*p*-value
Os10g0409400	Polygalacturonase isoenzyme 1 beta subunit precursor.	1407.258	0.00428
Os03g0291500	gb|Oryza sativa Japonica Group	1069.339	0.00028
Os07g0529000	Isocitrate lyase (Fragment).	850.443	0.00132
Os04g0659300	Protein of unknown function DUF26 domain containing protein.	672.462	0.00071
Os12g0437800	MPI	405.826	0.00242
Os03g0431600	Hypothetical conserved gene.	394.661	0.02582
Os06g0474800	Molecular chaperone, heat shock protein, Hsp40, DnaJ domain containing protein.	362.417	0.07243
Os02g0240100	Peroxidase 2	352.382	0.00018
Os07g0258400	OsNramp1 (Integral membrane protein).	335.331	0.09016
Os03g0765400	Conserved hypothetical protein.	330.079	0.00012
Os03g0291500	gb|Oryza sativa Japonica Group	1069.339	0.00028
Os06g0561000	Myo-inositol oxygenase.	310.050	0.00000
Os03g0826800	Unknown	236.045	0.00048
Os03g0277600	(Clone wusl1032) mRNA sequence.	227.549	0.00004
Os01g0702000	Protein of unknown function DUF151 domain containing protein.	217.955	0.00544
Os12g0236100	Unknown	211.464	0.05036
Os07g0142100	Unknown	204.823	0.06578
Os04g0688300	Haem peroxidase, plant/fungal/bacterial family protein.	196.887	0.00047
Os03g0184100	Hypothetical protein.	188.133	0.00528
Os03g0288000	Similar to Metallothionein.	185.139	0.00001
Os06g0549900	FAD linked oxidase, N-terminal domain containing protein	184.779	0.00471
Os09g0498500	CI260136 Oryza sativa (japonica cultivar-group) same as Lib 23(supermix) Oryza sativa (japonica cultivar-group) cDNA clone 045-M068R-B05 3', mRNA sequence [CI260136]	179.251	0.00002

Os08g0466600	YS1-like metal-nicotianamine transporter - Oryza sativa subsp.	177.707	0.05123
Os01g0494300	Hypothetical conserved gene.	169.737	0.02080
Os07g0179700	Rep: DD1A protein-like - Oryza sativa subsp. japonica (Rice)	168.124	0.00005
Os01g0733200	Heat shock transcription factor 29 (Fragment).	166.434	0.00265
Os12g0258700	Cupredoxin domain containing protein.	163.987	0.00591
Os09g0243200	Zinc finger, RING/FYVE/PHD-type domain containing protein	163.339	0.00768
Os07g0684100	Thioredoxin-like 1.	160.196	0.00274
Os09g0498400	Non-protein coding transcript.	157.287	0.00014
Os11g0262600	Conserved hypothetical protein.	154.345	0.04357
Os07g0664600	Glucose/ribitol dehydrogenase family protein.	150.617	0.00023
Os01g0839900	Thaumatin, pathogenesis-related family protein.	139.597	0.00232
Os05g0575000	Predicted protein.	137.093	0.01839
Os10g0360100	Sugar transporter protein.	129.737	0.00187
Os04g0355900	Rep: Quercetin 3-O-glucoside-6"-O-malonyltransferase-like - Oryza sativa subsp. japonica (Rice), partial (6%)	126.173	0.00318
Os11g0673200	Auxin-induced beta-glucosidase.	119.636	0.00448
Os01g0963000	Peroxidase BP 1 precursor.	118.409	0.00502
Os01g0644000	Twin-arginine translocation pathway signal domain containing protein.	116.208	0.00034
Os07g0683200	OsNAC6 protein.	115.376	0.00102
Os04g0390100	Heavy metal transport/detoxification protein domain containing protein.	114.931	0.00054
Os12g0550800	cDNA clone:001-040-C10, full insert sequence.	110.269	0.00227
Os01g0647200	Non-protein coding transcript.	107.906	0.02018
Os08g0473900	Alpha-amylase isozyme 3D precursor (1,4-alpha-D-glucan glucanohydrolase).	106.267	0.00171
Os08g0495800	Alkaline alpha galactosidase 3.	105.497	0.00171

Os05g0231700	Tonoplast membrane integral protein ZmTIP4-2.	104.767	0.01658
Os03g0183000	Hypothetical conserved gene.	98.393	0.00063
Os12g0448900	Pathogen-inducible alpha-dioxygenase.	97.490	0.01621
Os02g0191300	Amino acid transporter-like protein.	97.323	0.00082
Os01g0615100	Substilin /chymotrypsin-like inhibitor (Proteinase inhibitor).	93.661	0.01025

Supplemental Table S4 Top 50 genes downregulated in shoots of *ospitp6-3* seedlings.

Locus ID	Description	Fold	*p*-value
Os01g0975900	Tonoplast membrane integral protein ZmTIP1-2.	0.011	0.00901
Os01g0963600	ABA/WDS induced protein family protein.	0.016	0.05885
Os05g0217700	BURP domain containing protein.	0.020	0.01406
Os06g0253100	Heat shock protein Hsp20 domain containing protein.	0.020	0.08383
Os05g0579600	Homeodomain-like containing protein.	0.021	0.03064
Os03g0782200	Conserved hypothetical protein.	0.022	0.00381
Os03g0843800	Methyl chloride transferase.	0.025	0.03782
Os03g0228200	Conserved hypothetical protein.	0.027	0.03259
Os05g0462000	Rep: Chromosome chr8 scaffold_29, whole genome shotgun sequence - Vitis vinifera (Grape), partial (67%)	0.032	0.01862
Os01g0910900	Unknown	0.032	0.00166
Os03g0757200	UDP-glucuronosyl/UDP-glucosyltransferase family protein.	0.032	0.02015
Os06g0141200	RNA-binding protein EWS.	0.035	0.00930
Os12g0628100	Actin-depolymerizing factor 6 (ADF-6) (AtADF6).	0.035	0.01995
Os03g0797300	Rep: Os03g0797500 protein - Oryza sativa subsp. japonica (Rice), partial (57%)	0.036	0.00275
Os05g0508400	Mannose-binding lectin domain containing protein.	0.040	0.00256
Os10g0530500	Glutathione-S-transferase Cla47.	0.042	0.01691
Os01g0823600	Conserved hypothetical protein.	0.043	0.04843
Os03g0161900	Isoform 2 of Heat stress transcription factor A-2d.	0.045	0.06738
Os08g0559800	Hypothetical conserved gene.	0.048	0.22395
Os07g0412100	RNA-binding protein EWS.	0.049	0.01796
Os09g0478300	Conserved hypothetical protein.	0.049	0.02505

Os04g0444800	Ferric reductase-like transmembrane component family protein.	0.049	0.01905
Os04g0136600	Conserved hypothetical protein.	0.050	0.00015
Os05g0111300	B22EL8 protein.	0.051	0.00028
Os04g0607500	Oryza sativa high-affinity K+ transporter (HKT) 1;1., Sodium ion transport., Ion transporter.	0.054	0.00212
Os07g0630800	Nodule-enhanced malate dehydrogenase.	0.054	0.01531
Os05g0548900	Phosphoethanolamine methyltransferase.	0.054	0.02030
Os05g0215000	BURP domain containing protein.	0.054	0.01722
Os08g0139700	terpene synthase 6.	0.056	0.00543
Os03g0254200	Non-protein coding transcript.	0.056	0.04356
Os05g0586600	SIGE (RNA polymerase sigma subunit E); DNA binding / DNA-directed RNA polymerase/ sigma factor/ transcription factor.	0.057	0.05439
Os08g0374000	Bet v I allergen family protein.	0.057	0.08320
Os02g0629200	HvPIP2;1 protein.	0.057	0.01520
Os06g0120200	SAM dependent carboxyl methyltransferase family protein.	0.057	0.01118
Os02g0666200	Aquaporin.	0.058	0.01267
Os06g0593800	UDP-glucuronosyl/UDP-glucosyltransferase family protein.	0.058	0.00521
Os05g0217800	BURP domain-containing protein 2.	0.059	0.01790
Os05g0113400	Actin-depolymerizing factor 2 (ADF 2).	0.059	0.02409
Os05g0102000	SAM dependent carboxyl methyltransferase family protein.	0.059	0.00068
Os07g0464200	Oryza sativa Japonica Group Os07g0464200 (Os07g0464200) mRNA, partial cds	0.059	0.04412
Os07g0425000	Biopterin transport-related protein BT1 family protein.	0.059	0.03423
Os04g0657300	Oryza sativa Japonica Group cDNA, clone: J065196L10, full insert sequence	0.060	0.04530
Os10g0533500	Beta-ring hydroxylase (Fragment).	0.063	0.00972

Os06g0143400	Acyl-ACP thioesterase (Fragment).	0.063	0.00794
Os01g0151700	Short-chain dehydrogenase Tic32.	0.064	0.02155
Os06g0160700	Starch synthase I, chloroplast precursor (EC 2.4.1.21) (Soluble starch synthase 1) (SSS 1).	0.064	0.03278
Os04g0650700	OSIGBa0113E10.3 protein.	0.065	0.00030
Os01g0585300	Protein of unknown function DUF1118 family protein.	0.066	0.01375
Os02g0106100	Fructosyltransferase.	0.066	0.00004
Os01g0229600	Conserved hypothetical protein	0.066	0.00794

Supplemental Table S5 Top 50 genes upregulated in roots of *ospitp6-3* seedlings.

Locus ID	Description	Fold	*p*-value
Os07g0604600	B12D family protein.	316.496	0.00037
Os05g0170200	Conserved hypothetical protein.	163.622	0.00040
Os05g0469800	Pyruvate decarboxylase (EC 4.1.1.1) (Fragment).	154.278	0.01233
Os03g0431600	Hypothetical conserved gene.	142.086	0.00000
Os04g0516600	Pyridoxal phosphate-dependent transferase, major region, subdomain 1 domain containing protein.	124.297	0.00291
Os02g0585700	Oryza sativa (indica cultivar-group) cDNA clone: OSIGCRA221I19, full insert sequence	114.264	0.05341
Os05g0469600	Pyruvate decarboxylase (Fragment).	111.656	0.04021
Os03g0226200	Non-symbiotic hemoglobin 2 (rHb2) (ORYsa GLB1b).	110.775	0.00082
Os12g0437800	Similar to MPI.	110.018	0.00001
Os07g0511100	Oryza sativa Japonica Group Os07g0511100 (Os07g0511100) mRNA, complete cds	107.399	0.00000
Os01g0185400	WD40 repeat-like domain containing protein.	102.194	0.00053
Os01g0348900	SalT gene product (Salt-induced protein).	94.752	0.00103
Os07g0115200	Conserved hypothetical protein.	90.930	0.00021
Os10g0407200	Protein of unknown function DUF962 family protein.	90.604	0.00260
Os07g0162600	Alpha/beta hydrolase fold-3 domain containing protein.	85.435	0.38932
Os10g0360100	Sugar transporter protein.	84.725	0.00165
Os05g0375400	Beta-glucanase precursor.	80.459	0.00003
Os01g0595600	Alpha/beta hydrolase fold-1 domain containing protein.	79.419	0.00029
Os02g0586900	Hypothetical conserved gene.	78.591	0.00129

Os06g0506600	Ubiquitin-conjugating enzyme E2-17 kDa 8 (EC 6.3.2.19) (Ubiquitin- protein ligase 8) (Ubiquitin carrier protein 8) (UBCAT4A).	73.269	0.00049
Os04g0316200	Oryza sativa Japonica Group Os04g0322100 (Os04g0322100) mRNA, complete cds	69.681	0.00005
Os07g0125000	Pathogenesis-related protein PR-1 precursor.	68.917	0.00002
Os03g0826800	Unknown	68.856	0.00100
Os04g0650700	L-asparaginase (L-asparagine amidohydrolase).	68.622	0.00147
Os12g0242100	Glycine-rich cell wall structural protein 1 precursor.	68.216	0.00058
Os10g0370500	Glycoside hydrolase, catalytic core domain containing protein.	66.763	0.00141
Os11g0210500	Alcohol dehydrogenase.	66.015	0.00169
Os02g0802500	H(+)-translocating (Pyrophosphate-ENERGIZED) inorganic pyrophosphatase beta-1 polypeptide (EC 3.6.1.1) (Fragment).	63.840	0.00105
Os02g0168100	4-hydroxyphenylpyruvate dioxygenase (EC 1.13.11.27) (4HPPD) (HPD) (HPPDase).	63.029	0.00179
Os09g0364400	Oryza sativa Japonica Group cDNA, clone: J043034J15, full insert sequence	62.018	0.00414
Os03g0293500	Pyruvate decarboxylase isozyme 3 (EC 4.1.1.1) (PDC) (Fragment).	60.439	0.00033
Os11g0210300	Alcohol dehydrogenase 1.	57.094	0.01279
Os12g0562000	Hypothetical gene.	56.661	0.00164
Os06g0561000	Myo-inositol oxygenase.	56.376	0.00001
Os03g0113900	Protein of unknown function DUF584 family protein.	56.204	0.00026
Os04g0322100	Protein of unknown function DUF26 domain containing protein.	56.169	0.00000
Os10g0463800	Conserved hypothetical protein.	56.030	0.00132

Os02g0582600	Heavy metal transport/detoxification protein domain containing protein.	55.740	0.00051
Os08g0190100	Germin-like protein 8-7.	54.885	0.00026
Os03g0277700	Protein of unknown function DUF26 domain containing protein.	53.812	0.00043
Os10g0109600	Peroxidase (EC 1.11.1.7).	52.864	0.00026
Os04g0468600	Heavy metal transport/detoxification protein domain containing protein.	52.156	0.00245
Os04g0659300	Protein of unknown function DUF26 domain containing protein.	52.023	0.00081
Os03g0115800	Conserved hypothetical protein.	51.790	0.00055
Os02g0541300	Unknown	51.419	0.00082
Os07g0106200	Hexose transporter.	51.345	0.00027
Os11g0666000	Hypothetical gene.	50.104	0.00003
Os12g0222000	CI393477 Oryza sativa (japonica cultivar-group) seed Oryza sativa (japonica cultivar-group) cDNA clone J04B3014I11M3 3', mRNA sequence	49.864	0.00043
Os07g0638400	1-Cys peroxiredoxin.	48.306	0.00000
Os05g0158600	OsGA2ox1.	48.208	0.32444

Supplemental Table S6 Top 50 genes downregulated in roots of *ospitp6-3* seedling.

Locus ID	Description	Fold	*p*-value
Os10g0469100	Conserved hypothetical protein.	0.009	0.27120
Os01g0517800	Hypothetical conserved gene.	0.015	0.26919
Os08g0254500	Preprotein translocase subunit secY, chloroplastic.	0.015	0.26665
Os12g0165900	Rep: Os12g0165900 protein - Oryza sativa subsp. japonica (Rice), complete	0.022	0.26665
Os12g0515400	Plastidic 2-oxoglutarate/malate transporter.	0.036	0.45155
Os08g0323400	Rubredoxin (Rd).	0.037	0.05598
Os01g0600900	Chlorophyll a-b binding protein 2, chloroplastic.	0.039	0.21704
Os11g0176200	Predicted protein.	0.042	0.63516
Os11g0598700	tRNA pseudouridine synthase B, N-terminal domain containing protein.	0.044	0.27173
Os04g0602600	Pentatricopeptide repeat domain containing protein.	0.047	0.26928
Os05g0111300	B22EL8 protein.	0.048	0.02294
Os01g0974200	Metallothioneein.	0.056	0.03397
Os04g0669800	Methylthioribose kinase (EC 2.7.1.100).	0.062	0.28091
Os10g0452900	BR050002001C02.ab1 OC Oryza sativa cDNA clone BR050002001C02.ab1 similar to No protein aligment, mRNA sequence	0.063	0.04885
Os03g0138100	Nucleotide-binding, alpha-beta plait domain containing protein.	0.063	0.28427
Os03g0146800	Non-protein coding transcript.	0.066	0.04002
Os05g0227600	Proline-rich protein.	0.073	0.00033
Os07g0624600	Trehalose-6-phosphate phosphatase.	0.076	0.25358
Os04g0680400	Allantoinase (EC 3.5.2.5).	0.077	0.27449
Os03g0307200	Nicotianamine synthase 2 (EC 2.5.1.43) (S-adenosyl-L-methionine:S- adenosyl-L-methionine:S-adenosyl-methionine 3-amino-3- carboxypropyltransferase 2) (OsNAS2).	0.081	0.01194

Os07g0289800	Conserved hypothetical protein.	0.090	0.26343
Os05g0550300	Lipid transfer protein (Fragment).	0.098	0.00812
Os01g0188100	sopenicillin N synthase family protein.	0.101	0.29320
Os05g0531200	Pollen Ole e 1 allergen and extensin domain containing protein.	0.108	0.00431
Os02g0258200	High mobility group, HMG1/HMG2 domain containing protein.	0.115	0.02193
Os10g0452500	AU173297 Rice root Oryza sativa (japonica cultivar-group) cDNA clone R2062, mRNA sequence	0.122	0.02380
Os02g0653200	Cupredoxin domain containing protein.	0.127	0.00223
Os04g0366000	OSIGBa0110B10.6 protein.	0.127	0.24980
Os02g0658100	Tonoplast intrinsic protein2.	0.128	0.02609
Os03g0297900	AU032995 Rice shoot Oryza sativa (japonica cultivar-group) cDNA clone S0804_6Z, mRNA sequence	0.132	0.01226
Os02g0131800	Root-specific metal transporter.	0.140	0.01411
Os03g0134900	Glutathione S-transferase GSTF14 (Fragment).	0.142	0.00239
Os07g0631500	Hypothetical gene.	0.144	0.31874
Os01g0963600	ABA/WDS induced protein family protein.	0.153	0.01813
Os02g0662000	RCc3 protein.	0.154	0.00841
Os05g0563600	FAS1 domain domain containing protein.	0.157	0.00841
Os12g0274700	Petunia ribulose 1,5-bisphosphate carboxylase small subunit mRNA (clone pSSU 51), partial cds. (Fragment).	0.158	0.12388
Os09g0469600	Oryza sativa (indica cultivar-group) cDNA clone: OSIGCSN050H21, full insert sequence	0.160	0.00259
Os01g0756900	Aldehyde dehydrogenase, conserved site domain containing protein.	0.162	0.00396
Os02g0653000	Conserved hypothetical protein.	0.162	0.00041
Os01g0502700	Rep: Probable histone H2A.5 - Oryza sativa subsp. indica (Rice), partial (72%)	0.164	0.03341
Os10g0536700	Peroxidase 1.	0.165	0.00210

Os10g0453000	Glycine-rich cell wall structural protein 2 (Fragment).	0.166	0.02831
Os09g0484200	Conserved hypothetical protein.	0.167	0.00683
Os06g0160100	Unknown	0.169	0.03283
Os04g0496300	Conserved hypothetical protein.	0.178	0.00495
Os05g0382600	Annexin-like protein RJ4.	0.179	0.00701
Os10g0534500	Similar to Resistance protein candidate (Fragment). [Os10t0534500-01]	0.180	0.72286
Os02g0662000	RCc3 protein.	0.184	0.01813
Os12g0274700	Similar to Petunia ribulose 1,5-bisphosphate carboxylase small subunit mRNA.	0.186	0.07049

Supplemental Table S7 Actual values of signal intensities for expression levels of genes associated with Pi uptake, translocation, and signaling in shoots of WT and *ospitp6-3* seedlings.

gene	gene ID	signal intensity[a]		*ospitp6*/WT
		WT	*ospitp6*	
PT1	Os03g0150600	3749.25	5896.75	1.5192
PT2	Os03g0150800	73	64.75	1.161
PT3	Os10g0444600	696	669.25	0.9616
PT4	Os04g0186400	2654.25	2495	0.4669
PT5	Os04g0185600	54	55	1.0185
PT6	Os08g0564000	120.5	111.5	1.0045
PT7	Os03g0136400	54.5	54.5	1
PT8	Os10g0444700	185	80.5	0.4108
PT9	Os06g0324800	52	50.5	0.9712
PT10	Os06g0325200	56.5	53.5	0.9469
PT11	Os01g0657100	53	50.75	1.0025
PT12	Os03g0150500	110.5	70.75	0.6403
PT13	Os04g0186800	66.75	63	0.9438
PT14	Os02g0593500	14204.5	2181.75	0.1395
PT15	Os02g0767500	3565.75	5474.25	1.5389
PT16	Os03g0263400	1024.75	716.75	0.6994
PT17	Os04g0448800	84.75	98.75	1.6036
PT18	Os06g0210500	163	531	3.2577
PT19	Os09g0454600	385.25	679.5	1.7638
PT20	Os09g0554000	100	113.25	1.1325
PT21	Os01g0279700	17273.25	13502	0.7258
PT22	Os01g0852200	205	946	4.0462
PT23	Os05g0451100	145.25	337	2.3201
PT24	Os09g0570400	622.25	554.25	0.8701
PT26	Os12g0180100	180	393.75	2.2223
PHF1	Os07g0187700	3241.75	7581.25	2.3386
PHF1L	Os03g0799900	432.5	492.25	1.1382
PHO1;1	Os01g0110100	559.25	77.5	0.12
PHO1;2	Os02g0809800	182.75	73.75	0.4036

PHO1;3	Os06g0493600	699	87.5	0.1252
PHR1	Os03g0329900	774.25	1298.5	1.6133
PHR2	Os07g0438800	71	74	1.0801
PHR3	Os02g0139000	649.75	3000	4.6172
IPS1	Os03g0146800	167.5	56.25	0.5718
PHL3	Os09g0299000	1726	2387	1.383
SQD1	Os05g0387200	56	56	1.2922
OsPAP1	Os08g0378900	1110.5	2534.75	2.4156
SPX1	Os06g0603600	1826	1626.25	1.0974
SPX2	Os02g0202200	754.5	413.25	0.5477
SPX3	Os10g0392600	53.25	56.5	1.061
SPX5	Os03g0406100	67	61	0.9104
SPX6	Os07g0614700	350.75	762.75	2.1746
FCO11	Os04g0555300	12717.75	15303	1.0132
TPT2	Os05g0241200	1168	701.25	0.6385

[a]Averages of actual values obtained with 4 biological replicates in DNA microarray analysis.

Supplemental Table S8 Actual values of signal intensities for expression levels of genes associated with Pi uptake, translocation, and signaling in roots of WT and *ospitp6-3* seedlings.

gene	gene ID	signal intensity[a]		*ospitp6*/WT
		WT	*ospitp6*	
PT1	Os03g0150600	1264	1572.5	0.9756
PT2	Os03g0150800	2577.25	1682.25	1.0833
PT3	Os10g0444600	170.5	97.5	0.5718
PT4	Os04g0186400	388.25	244.5	0.9694
PT5	Os04g0185600	170.75	94.75	0.5549
PT6	Os08g0564000	177	167.5	0.7498
PT7	Os03g0136400	216	75.75	0.619
PT8	Os10g0444700	204.5	106	0.8583
PT9	Os06g0324800	193	101.75	0.5272
PT10	Os06g0325200	228.75	112.5	0.4918
PT11	Os01g0657100	158.25	91.5	0.396
PT12	Os03g0150500	129.75	90.75	0.6994
PT13	Os04g0186800	369.5	159	0.4303
PT14	Os02g0593500	279	63	0.2331
PT15	Os02g0767500	519.5	951	1.8919
PT16	Os03g0263400	82.25	303	3.6839
PT17	Os04g0448800	87.5	81	1.9176
PT18	Os06g0210500	146.25	199.75	1.3658
PT19	Os09g0454600	255.75	621	2.4282
PT20	Os09g0554000	137	121.5	0.8869
PT21	Os01g0279700	86.75	226.25	1.6693
PT22	Os01g0852200	155.5	271.5	1.9453
PT23	Os05g0451100	544	3219.5	5.9182
PT24	Os09g0570400	80	176	2.9984
PT26	Os12g0180100	91.75	114	1.1954
PHF1	Os07g0187700	576.25	2685	4.6594
PHF1L	Os03g0799900	77.5	151	1.9484
PHO1;1	Os01g0110100	569.75	154.5	0.2987
PHO1;2	Os02g0809800	1065	298	0.2798

PHO1;3	Os06g0493600	247.75	83	0.335
PHR1	Os03g0329900	211.5	714.5	4.5074
PHR2	Os07g0438800	81	73	3.731
PHR3	Os02g0139000	169	1779.75	10.5311
IPS1	Os03g0146800	1692.75	110.5	0.1875
PHL3	Os09g0299000	171	538.5	3.1491
SQD1	Os05g0387200	186	98.5	2.196
OsPAP1	Os08g0378900	124.5	276	1.6331
SPX1	Os06g0603600	321	485	1.9316
SPX2	Os02g0202200	175	465.25	2.6586
SPX3	Os10g0392600	242	126	0.5207
SPX5	Os03g0406100	135.5	102.75	0.7583
SPX6	Os07g0614700	139.25	331	2.377
FCO11	Os04g0555300	597.25	740.75	1.2635
TPT2	Os05g0241200	108.5	102.25	0.7795

[a]Averages of actual values obtained with 4 biological replicates in DNA microarray analysis.

Supplemental Table S9 Primers used in this study.

Gene	Gene ID	Forward primer (5'→3')	Reverse primer (5'→3')
For cDNA cloning			
AtPITP7	AT5G63060	CACCATGTCGATTCGTTTCAGCTCAA	TTAGCTCCGGAAATTTGATGGTAATG
OsPITP6 (for *Ubi:OsPITP6*)	Os02g0321500	AAAGTCGACATGGCTGCAGCTTGCTC	AAAAGGCCTTAATTGCGGAAATCAGGAG
OsPITP6 (for *HBT:OsPITP6:GFP*)	Os02g0321500	AAAGGATCCATGGCTGCAGCTTGCTC CCTTC	GGAAGGCCTCTAATTGCGGAAATCAGGAGGTACAG
For cloning of promoter regions			
WS.0-type *AtPITP7*	AT5G63060	GTCCTGCAGTTAAGATCGATGC	GTCGTCGACTGACTACAAGGAGTAA
Sorbo-type *AtPITP7*	AT5G63060	ATTCTGCAGCGGAGTTTTCGA	GTCGTCGACTGACTACAAGGAGTAAAAA
Col.0-type *AtPITP7*	AT5G63060	GTCCTGCAGAATATTAGTACGTAGAGTATTC	GTCGTCGACTGACTACAAGGAGTAA
Rsch-4-type *AtPITP7*	AT5G63060	GTCCTGCAGTCTTTGAACTAGTCTGC	GTCGTCGACTGACTACAAGGAGTAA
Ler.0-type *AtPITP7*	AT5G63060	GTCCTGCAGAGTAATATTAGTACGTAGAGTATTC	GTCGTCGACTGACTACAAGGAGTAA
Old-1-type *AtPITP7*	AT5G63060	ATTCTGCAGGATGCAGCATACA	GTCGTCGACTGACTACAAGGAGTAAAA
For qPCR			
AtPITP7	AT5G63060	GGGCTGCTTGATCCAATAGA	CGAATCCACGGAGATCAAAT
GAPDH	AT1G16300	TTGGTGACAACAGGTCAAGCA	AAACTTGTCGCTCAATGCAATC
ACT2	AT3G18780	TGGGATGAACCAGAAGGATG	AAGAATACCTCTCTTGGATTGTGC

OsPITP6	Os02g0321500	CGAGGATTTCAGGTGGAAAA	ACAAACGGTGCATCAACAAA
OsUBQ5	Os01g0328400	ACCACTTCGACCGCCACTACT	ACGCCTAAGCCTGCTGGTT
OsACD1	Os03g0146400	GTGTTGCCTTCCACTGTCCT	ACTGAACATCCGCAGGAATC
OsNAC4	Os01g0816100	CGGAAATAGGAGTGATGGCTAGA	ACCACATTTGCAGAATCATGCT
OsPHO1;1	Os01g0110050	TCAAGTACCGGGACGTCTTC	AGAACAGCAGCAGCAGGAAC
OsPHO1;2	Os02g0809700	CGATACTGCTTCATGCGTGT	ACGCTGTAAATTCCATGTGC
OsPHO1;3	Os06g0493600	TGGCTACTGCACCAGAGTGA	TGATTTATGTCCCCCTCGTC
OsPT9	Os06g0324800	CTTCTTCTCCGCCAACCTC	CACAGGAAGCCGATGGAG
OsPT10	Os06g0325200	CCTCCTCGACATCCCCTACT	ATGACGGCCTGGAACTTG
OsPT14	Os02g0593500	CAAATGCTCAGCAAGTTGGA	TGAAGCATGCTGACAAGACC
